# Effects of vitamin B_12_ supply on cellular processes of the facultative vitamin B_12_ consumer *Vibrio campbellii*

**DOI:** 10.1128/aem.01422-24

**Published:** 2025-01-22

**Authors:** Luna-Agrippina Groon, Stefan Bruns, Leon Dlugosch, Heinz Wilkes, Gerrit Wienhausen

**Affiliations:** 1 Institute for Chemistry and Biology of the Marine Environment (ICBM), School of Mathematics and Science, Carl von Ossietzky Universität Oldenburg 436553 , Oldenburg, Germany; University of Illinois Urbana-Champaign, Urbana, Illinois, USA

**Keywords:** vitamin B_12_, autoinducer-2, methionine, *Vibrio campbellii*, facultative vitamin B_12 _consumer, virulence factor, *Vibrionales*, *Gammaproteobacteria*

## Abstract

Vitamin B_12_ (cobalamin, herein B_12_) is a key cofactor for most organisms being involved in essential metabolic processes. In microbial communities, B_12_ is often scarce, largely because only few prokaryotes can synthesize B_12_ *de novo* and are thus considered B_12_-prototrophs. B_12_-auxotrophy is mostly manifested by the absence of the B_12_-independent methionine synthase, MetE. Here, we focus on bacteria that we classified as facultative B_12_ consumers as they encode both B_12_-independent and -dependent (MetH) methionine synthases yet largely cannot synthesize B_12_ *de novo*. The genus *Vibrio* belongs to this group, and our work shows that upon B_12_ supply growth of *Vibrio campbellii* is accelerated and autoinducer-2 (AI-2) concentrations are enhanced. We speculate that methionine synthesis efficiency, dependent on B_12_ availability, is linked to AI-2 synthesis. The precursor for AI-2 synthesis is S-adenosyl-L-homocysteine (SAH), which in turn requires methionine as a precursor. In almost all *Vibrio* species studied, *btuF* (B_12_-binding protein), which is required for B_12_ uptake, and *cobD* (Adenosylcobinamide-phosphate synthase), which enables remodeling of B_12_-like compounds, are encoded on the same operon as *pfs* (or *mtnN*, Adenosylhomocysteine nucleosidase), the first enzyme in the two-step AI-2 synthesis reaction. Transcriptomic analyses show that virulence factors, such as toxin synthesis, fimbriae formation, and activation of the type-6 secretion system, which have been shown to be regulated by quorum sensing via AI-2, are significantly upregulated in *V. campbellii* when B_12_ is available. Our results demonstrate that *V. campbellii* is a facultative B_12_ consumer and indicate that B_12_ availability affects AI-2 levels and thus potentially virulence factor regulation.

**IMPORTANCE:**

Metabolites play a key role in microbial metabolism and communication. While vitamin B_12_ is an essential cofactor for important enzymatic reactions, autoinducer-2 mediates interspecies signaling and can regulate the expression of genes that are crucial for virulence and survival. In our study, we hypothesize and present findings how these two important metabolites are linked in *Vibrio* species. *Vibrio campbellii* grows without B_12_ but at an accelerated rate when B_12_ is present, and we detect higher AI-2 values in cultures with B_12_ amendment. Transcriptome analyses show how vitamin B_12_ availability significantly upregulates gene expression of virulence factors such as toxin synthesis, fimbrial formation, and activation of the type-6 secretion system in *V. campbellii*.

## INTRODUCTION

Cobamides, a group of B_12_-like analogs that carry cobalt in the center of their corrinoid ring but differ in the attached lower ligand, are essential coenzymes for the majority of all living organisms. Vitamin B_12_ (cobalamin, herein B_12_) is the most commonly found cobamide that is bioavailable to most organisms as a coenzyme. A distinctive feature of cobamides is that only prokaryotes encode the genetic ability to synthesize these complex metabolites, which have a size of at least 1,200 Dalton (1). Despite a high B_12_ dependence of many organisms, genome analyses of prokaryotes from various habitats show that only a small percentage possess genes of the complete B_12_ pathway and can thus be classified as B_12_-prototrophs (2, 3) and therefore contribute to its availability in nature. For instance, while a large share of organisms within a human skin microbiome possess genes encoding B_12_-dependent enzymes, only a minority encode genes for *de novo* cobamide synthesis (4). Similar observations were obtained in a soil microbiome, where less than 10% of the prokaryotes encode genes for *de novo* cobamide synthesis (5). Moreover, the number of synthesized B_12_ molecules varies greatly among B_12_-producing bacteria, and a large fraction does not even share the coenzyme freely, which further limits B_12_ accessibility (6, 7).

In nature, B_12_ functions as a coenzyme or cofactor in a variety of reactions, including B_12_-dependent isomerases (1) and reductive dehalogenation (8), and can serve as a non-enzymatic transcriptional or translational regulator by binding to riboswitches (9, 10). However, the best-known function of B_12_ as coenzyme is methylation reactions (11), in which a methyl group is transferred from methylcobalamin to an acceptor molecule. Probably most bacterial B_12_ auxotrophies observed in nature can be explained by this reaction involving the B_12_-dependent methionine synthase (MetH). Methionine is a sulfur-containing essential proteinogenic amino acid and is also a precursor of other amino acids such as cysteine and taurine and the basis for the synthesis of the antioxidant glutathione and S-adenosyl-methionine (12).

Instead of MetH, some prokaryotes possess the B_12_-independent methionine synthase MetE. Hence, they can thrive without *de novo* B_12_ synthesis or its uptake and thus can neither be classified as B_12_ auxotrophic nor B_12_ prototrophic. However, the effective rate of methionine synthesis by MetE can be significantly lower than that of B_12_-dependent MetH (13, 14). Thus, it is not surprising that some organisms encode both the *metH* and *metE* that enable methionine synthesis either with or without available B_12_ and therefore represent a group that we herein refer to as facultative vitamin B_12_ consumers. A representative of this group is *Vibrio campbellii*, a marine bacterium that is pathogenic to some marine eukaryotic organisms (15, 16). The pathogenicity of *V. campbellii* was shown to be linked to quorum sensing (QS), as the virulence of the wild type compared to mutants lacking the QS compounds autoinducer-1 (AI-1) and autoinducer-2 (AI-2) was significantly higher (16, 17). The synergistic function of AI-1 and AI-2 in the regulation of virulence genes has also been shown for the highly virulent human pathogen *Vibrio cholerae* (18, 19). However, this observation does not only apply to *V. campbellii* and *V. cholerae*, rather most pathogenic bacteria encode the genes for AI-2 synthesis (20). An effective and widespread virulence factor in *Vibrio* spp. is the type-6 secretion system (T6SS), by which effector proteins can be translocated into neighboring prokaryotic or eukaryotic cells in a contact-dependent manner (21). Regulatory mechanisms of the T6SS can be versatile and mostly integrate with existing regulatory pathways and signal transduction devices (21, 22). For *V. cholerae*, the HapR quorum-sensing regulator, which is controlled by AI-1 and AI-2 concentration, is proposed to have a regulatory effect on the T6SS (22, 23).

Interestingly, the basis of the AI-2 synthesis, as well as other QS compounds such as acyl-homoserine lactones (AHL), is methionine which is synthesized in the activated methyl cycle. Following the B_12_-dependent or -independent synthesis of methionine, the enzymes S-adenosylmethionine synthetase and DNA (cytosine-5)-methyltransferase synthesize S-adenosyl-L-homocysteine, which is the precursor to AHLs and AI-2. The conversion of S-adenosyl-L-homocysteine to L-homocysteine can occur in two different enzymatic reaction routes. First, the adenosylhomocysteine nucleosidase (PFs) and S-ribosylhomocysteine lyase (LuxS) enzymes synthesize L-homocysteine in a two-step reaction while simultaneously producing AI-2. A second alternative is the one-step reaction, involving S-adenosyl-L-homocysteine hydrolase that can catalyze the back reaction of S-adenosyl-L-homocysteine to L-homocysteine. *V. campbellii* exhibits the two-step reaction that leads to the synthesis of AI-2. While *V. campbellii* can synthesize methionine with both B_12_-independent and -dependent synthase, possibly affecting the efficiency of subsequent reactions in the activated methyl cycle, *V. campbellii* cannot synthesize B_12_ *de novo*. However, it possesses genes for lower ligand synthesis (LL) and for the nucleotide loop assembly (NLA) that enables remodeling of unsuitable cobamides, as has been shown for most *Vibrio* species (24). The species *V. cholerae*, which is related to *V. campbellii*, has been experimentally shown to remodel the lower ligand of pseudo-B_12_, which is unsuitable for its needs (25, 26).

While it is well known that B_12_ availability is critical for the synthesis of methionine in bacteria, we know little about other metabolic reactions or cellular processes affected by its availability so far. The fact that *V. campbellii* can grow both with and without the addition of B_12_ because it is neither a B_12_ prototroph nor an auxotroph, allows us to study the effects of B_12_ on profound cellular processes. Furthermore, synthesis of AI-2 has been identified predominantly in *Gammaproteobacteria* (20, 27); however, whether these bacteria can *de novo* synthesize B_12_ or require B_12_ for methionine synthesis has to the best of our knowledge not been addressed in detail.

We hypothesize that the accessibility of B_12_ has an effect on various cellular processes in *V. campbellii*, including the synthesis of methionine, which in turn could have a possible effect on the synthesis of AI-2.

## MATERIALS AND METHODS

### Cultivation conditions of growth experiments

Growth experiments with *V. campbellii* DSM 19270 were conducted in a modified minimal (AB)-medium (28) by using 2 mM (10 mM C) glutamate as the sole carbon source and omitting casamino acids, L-arginine, and glycerol. Prior to cultivation in minimal media, *V. campbellii* was inoculated in marine broth (MB) at 28°C and washed three times with AB-medium to omit any vitamin residues from MB cultivation. In the first experimental set-up, *V. campbellii* was cultured in AB-medium in test tubes in a volume of 10 mL with supplementations of B_12_ (1 nM; as cyanocobalamin), cobinamide (1 nM), or methionine (10 µM) at 20°C. In the following experiments, cultures of *V. campbellii* were grown in a volume of 1.4 L with the addition of B_12_ (1 nM; as cyanocobalamin) at 20°C. Each experiment was set up in biological triplicates and included a treatment without the addition of B_12_ or B_12_-like compounds and a sterile control without bacteria. All cultures were incubated shaking at 100 rpm in the dark, and growth was monitored by measuring the optical density (600 nm) and bacterial cell counts.

### Bacterial cell enumeration

Subsamples for bacterial cell enumeration were collected under sterile conditions, fixed with glutardialdehyde (Carl Roth, Germany) at a final concentration of 2%, incubated at 4°C for 30 minutes, and then stored at −20°C until analysis. SybrGreen I (Invitrogen, United Kingdom) as staining solution and TrueCountBeads (BD) for volume verification were added to samples which were then measured using a BD Accuri C6 cytometer (BD Biosciences, USA). Flow cytometer measurement was conducted as previously described (29).

### Autoinducer-2 bioassay

For the detection of AI-2, subsamples were collected at approximately 10 h intervals. The culture was filtered through a 0.22 µm polyethersulfone filter (Minisart, Sartorius, Göttingen, Germany) and stored at −20°C until analysis. The bioassay was conducted as described elsewhere (28) but modified by replacing casamino acids with tryptone.

Briefly, reporter strain BB170 was cultured overnight at 30°C until it showed bright luminescence and was then diluted 1:10,000 in fresh AB medium. To each well, 10 µL of the filtered sample was added to 90 µL of the culture of the reporter strain. Samples were measured in biological triplicates every 30 minutes for 7 hours in black 96-well plates (BRANDplates, Brand, Wertheim, Germany) using a TECAN plate reader. In addition, a positive control (cell-free exudate of AI-2-producing *Vibrio harveyi* strain BB152), culture exudate, and a negative control (AB medium) were measured in biological triplicates. Before each measurement, the plate was shaken to activate luminescence, which was then measured in counts per second. For analysis, a relative response ratio was calculated to account for interplate variation after the following equation:


$$Relative\;response\;ratio=\frac{\left(experimental\;sample\;ratio\right)-\left(negative\;control\;ratio\right)}{\left(positive\;control\;ratio\right)-\left(negative\;control\;ratio\right)}.$$

To be mentioned here is the fact that with the AI-2 bioassay, there can be variability due to inconsistent normalization between references by using different media matrices (30). Following the established AI-2 bioassay protocol of Taga (28), all cultivations were performed in AB-medium and were not compared with relative luminescence across different media (28).

### Detection of B_12_ biosynthesis and activated methyl cycle genes in bacterial genomes

Publicly available genomes were downloaded (accessed on 23 November 2022) from the Joint Genome Institute’s Integrated Microbial Genomes with Expert Review database (JGI/IMG/MER, https://img.jgi.doe.gov/cgi-bin/mer/main.cgi) (31). Pre-requirement for downloading genomes was sequencing status classified as “finished.” To verify the completeness of all genomes, we applied the approach as described elsewhere with slight modifications (2). Briefly, we removed all genomes with less than 54 housekeeping genes, applying a stringent selection and searched for 55 single housekeeping gene copies in all genomes (32, 33). In addition, we reduced multiple assigned species to a single genome, selecting for the species with the highest housekeeping gene numbers as representative. Strains with genus assignments but without species name assignments were considered as one group and reduced to one genome sp., also selected for the highest housekeeping gene numbers. The list of species was manually curated for species duplicates. Of all downloaded genomes (14,109), 3,716 genomes remained and were used for further analysis.

Cobalamin biosynthesis pathway genes, included in “KEGG-porphyrin and chlorophyll metabolism” and genes of the activated methyl cycle (*metH*, *metE*, *metK*, *DNMT*, *pfs*, *AHCY*, and *luxS*), were downloaded in IMG/MER comprising “KEGG-Function ID” and “EC-number” for each enzyme. To test whether identified B_12_ metabolism and activated methyl cycle enzymes are present in the 3,716 genomes, the “function profile: function vs genome” tool was applied. Genes were attributed into cobalamin pathway subgroups “anaerobic corrin ring synthesis (ANAE),” “aerobic corrin ring synthesis (AER),” “NLA,” and “LL.” Our criterion for genomes to feature the cobalamin synthesis subgroups was the minimum presence of genes as follows: ANAE (83%; 10/12), AER (90%, 9/10), NLA (80%; 4/5), and LL (75%; 3/4). Genomes were considered as “lacking B_12_ synthesis” when none of the criteria were met, “cobamide salvager” when genes for LL and NLA were present, vitamin B_12_ prototroph when NLA, LL, and at least AER or ANAE were present and corrin ring synthesizer when AEA or ANAE and NLA existed (Data S1).

### RNA extraction

For RNA extraction, subsamples were withdrawn under laminar flow in the early exponential phase and pelleted at 2,800 g for 5 min. After removal of the supernatant, the pellet was shock frozen in liquid nitrogen and stored at −80°C until further analysis. Cell pellets were thawed and mixed with 15 mg/mL lysosome and incubated at 20°C for 30 min. RNA was then isolated following the instructions given for the RNeasy kit (Qiagen, Hilden, Germany) and purified using the RNeasy MinElute Cleanup Kit (Qiagen).

### Library preparation, cDNA sequencing, and transcriptome analysis

The RNA sequencing analyses were carried out by DNASense (Aalborg, Denmark). In short, the concentration of RNA in each sample was measured in duplicate using the Qubit HS RNA assay. RNA quality and integrity were confirmed for each sample using TapeStation with RNA ScreenTape (Agilent Technologies). Depletion of the samples was performed via Illumina RiboZero Plus kit (Illumina). For sequencing preparation, the NEB Next Ultra II RNA library preparation kit (New England Biolabs) was used according to the manufacturer’s instructions. Library concentrations were measured with Qubit HS DNA assay, and library size was estimated with TapeStation D1000 ScreenTapes (Agilent Technologies). Finally, each sample was pooled in equimolar concentrations and paired-end sequenced (2 × 150 bp) on a NovaSeq 6000 system (Illumina, USA).

Illumina reads were quality checked, and low-quality regions, as well as adaptor sequences, were trimmed using Trimmomatic (34) 0.3648 (ADAPTER:2:30:10 SLIDINGWINDOW:4:25 MINLEN:100). Ribosomal RNA was depleted from high-quality reads using SortMeRNA (35) and nonredundant databases for 16S (90%id), 23S (98%id) (36), and 5S (98%id) (37). Resulting paired mRNA reads longer than 100 bp were mapped to genes of the genome from *V. campbellii* DSM 19270 using bowtie2 in more-sensitive-local mode (38). Differential gene expression between control and 1 nM B_12_ added was calculated using R v4.0.5 (R-Core-Team, 2022) and the DESeq2 package (39). Genes with a log2-fold change larger than 1.5 and smaller than −1.5 with a Benjamini-Hochberg (BH)-adjusted (40) *P*-value below 0.05 were considered significantly up-/downregulated.

### Data accession and deposition

Transcriptomic sequences generated in this study were deposited in European Nucleotide Archive (ENA) at EMBL-EBI with the accession number PRJEB66273, using the data brokerage service of the German Federation for Biological Data (GFBio), in compliance with the Minimal Information about any (X) Sequence (MIxS) standard (41, 42).

### Extraction and detection of B_12_ and respective building blocks

During mid (42–52 h), late exponential (52–66 h), and early stationary phase (76–90 h), subsamples were withdrawn for the analysis of intra- and extracellular B_12_ and α-ribazole. For extracellular B_12_, 150 mL of culture was filtered through a 0.22 µm bottle top filter (PES bottle-top filter, Nalgene Thermo Scientific) to gain cell-free exudate. For intracellular B_12_ and α-ribazole, 50 mL of culture was centrifuged at 3,550 g for 10 min forming a pellet. All samples were stored at −20°C until further extraction and analysis by liquid chromatography–mass spectrometry (LC-MS) as previously described (43, 44).

In short, extraction of extracellular B_12_ and α-ribazole from culture medium was conducted with a solid phase extraction column (Bond Elut PPL, 1 g, Agilent, Santa Clara, CA, USA), activated with 20 mL methanol and 20 mL H_2_O adjusted to pH 6 with hydrochloric acid (37%). Samples of the culture medium were adjusted to the same pH with hydrochloric acid (37%) and passed over the column. H_2_O (pH 6) was used to wash the remaining salt from the column, and the analytes were eluted with methanol. The solvent was evaporated under a flow of nitrogen gas, and the dry samples were further processed as described recently (45).

For intracellular analysis, pellets were resuspended in methanol and homogenized in a bead beater. The extraction was repeated twice. After centrifugation, the supernatant was separated from the bacterial cell pellet, and the solvent was evaporated under a stream of nitrogen gas. All other steps were conducted as described in reference 45. Briefly, filtered extracts were analyzed on a TSQ Quantum AM triple quadrupole mass spectrometer (Thermo Fisher Scientific) with heated electrospray ionization in positive mode (HESI+) as described elsewhere (46). In summary, source parameters were set as follows: spray voltage 3,000 V, vaporizer temperature 400°C, transfer tube temperature 340°C, sheath gas 60 arbitrary units, and auxiliary gas 20 arbitrary units. An Ultimate 3000 HPLC (Thermo Fisher Scientific) with a Kinetex Evo C18 column (100 × 2.1 mm, 2.6 µm pore size, Phenomenex, Torrance, CA, USA) was linked to the mass spectrometer to separate the individual analytes. Every sample had an injection volume of 5 µL. The following eluents were used: 10 mM ammonium formate (pH 6.0; A) and acetonitrile (B) with the following solvent gradient: 0–13 min 100%–75% A; 13–15 min 75%–0% A; 15–19 min 0% A; 19–21 min 0%–100% A; 21–26 min 100% A. Cobalamin was quantified by external calibration using commercially available standard compounds in combination with recovery experiments under the influence of matrix using seawater and bacterial samples spiked with cobalamin and processed in the same manner. Alpha-ribazole was synthesized as described in reference 46. The four forms of B_12_ (CB_12_, AB_12_, MB_12_, and HB_12_) were analysed intracellularly with recoveries between 74% and 99%. Alpha-ribazole had a recovery of 97%. Extracellularly, the four B_12_ forms had recoveries ranging between 48% and 91%, and α-ribazole had a recovery of 71%. The limits of detection for extracellular analysis were 0.08, 0.10, 0.05, and 6.15 pM for CB_12_, AB_12_, MB_12_, and HB_12_ and 0.03 pM for α-ribazole, respectively (Table S1).

## RESULTS

### Gene analyses of the activated methyl cycle and vitamin B_12_ pathway

**Fig 1:**
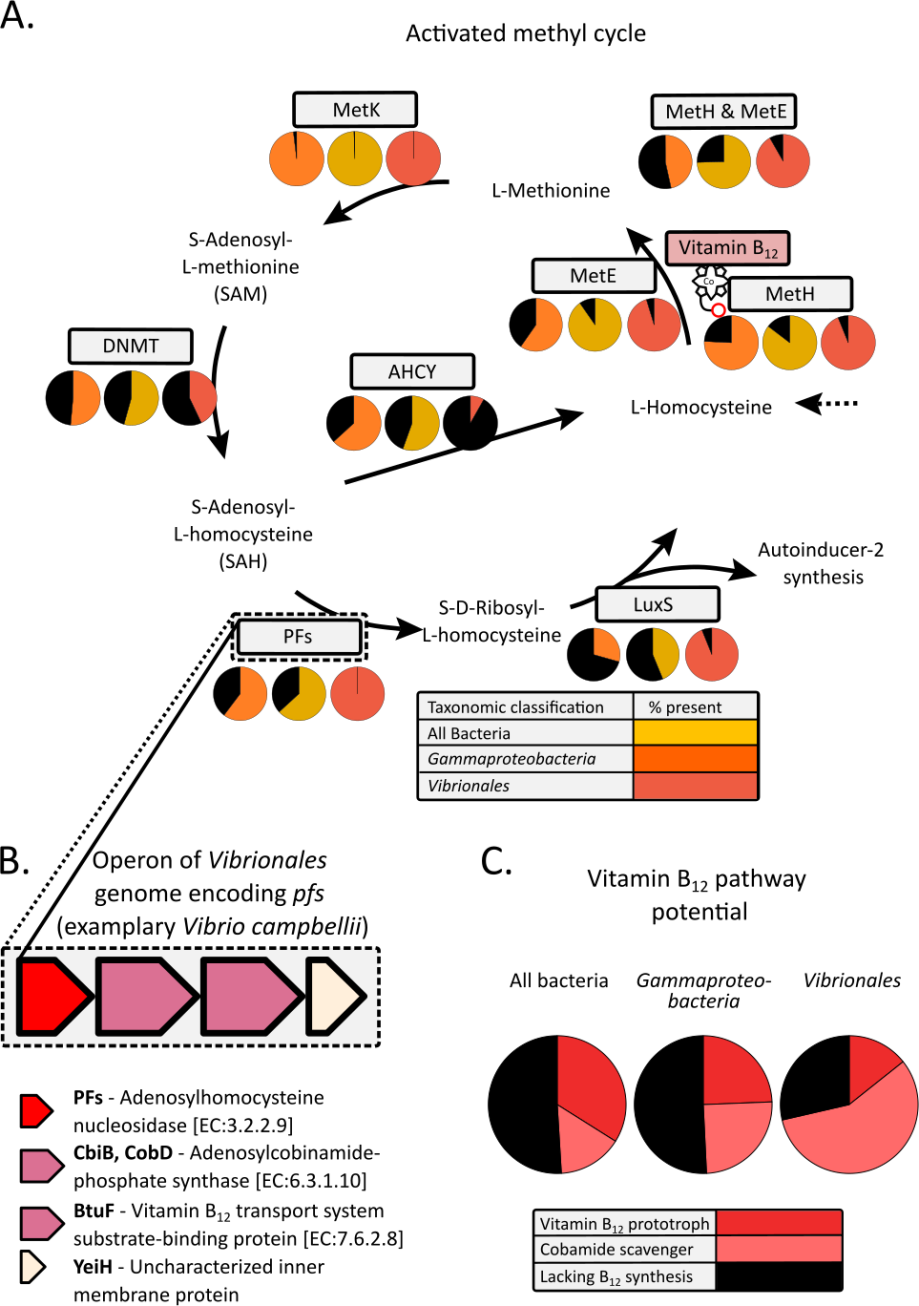
Genetic potential of the activated methyl cycle and description of the operon encoding *pfs*. (A) Illustrates the activated methyl cycle with description of the metabolic products and the enzymes (MetE, B_12_-independent methionine synthase; MetH, B_12_-dependent methionine synthase; MetK, S-adenosylmethionine synthetase; DNMT, DNA [cytosine-5]-methyltransferase 1; PFs, adenosylhomocysteine nucleosidase; LuxS, S-ribosylhomocysteine lyase) required for metabolic reactions. Below the enzymes (shown in gray boxes, for PFs in red) are pie charts showing percentages of bacteria (orange), *Gammaproteobacteria* (yellow), and *Vibrionales* (red) that encode the gene and percentages that do not (black). More detailed information on the presence of the genes can be obtained from Data S1. (B) Presentation of the operon on which *pfs* of *V. campbellii* is encoded with additional information on also encoded genes/enzymes. Details of other corresponding operons of other analysed *Vibrionales* spp. can be found in Fig. S1. (C) The pie charts display the percentage distribution of B_12_ biosynthesis potential of all bacteria, *Gammaproteobacteria*, and *Vibrionales* spp. analyzed. A distinction is made between B_12_ (cobamide) producer (red), lacking B_12_ biosynthesis (black) and cobamide scavanger/LL (pink). Details of the B_12_ biosynthesis potential of individual strains can be found in Data S1.

Bacteria that encode both B_12_-dependent (MetH) and B_12_-independent (MetE) methionine synthase constitute 47% of the 3,716 genomes evaluated. Considering only *Gammaproteobacteria*, the fraction is 75% and even 92% when considering only *Vibrionales*. About half of all bacterial genomes possess the *DNMT* gene, which is required for the catalytic reaction to convert S-adenosyl-L-methionine to SAH, with only minor differences between all bacterial groups analyzed. Thus, the genetic requirements for the one-step or two-step reaction that converts S-adenosyl-L-homocysteine into L-homocysteine differ distinctly. Almost all *Vibrionales* genomes examined exhibit *pfs* (100%) and *luxS* (94%), and only 8% possess the one-step reaction by means of the adenosylhomocysteinase (*AHCY*). On the other hand, fewer *Gammaproteobacteria* possess the two-step reaction (63% *pfs* and 44% *luxS*), yet 56% encode *AHCY*. Considering the total number of bacterial genomes analyzed, the share of bacteria possessing the two-step reaction (60% *pfs* and 29% *luxS*) is significantly lower compared to the one-step reaction (*AHCY*, 63%; Fig. 1A; Data S1)

We discovered a feature in 82% of all *Vibrionales* and some *Altermonadales* (not discussed in detail here), not observed in any other taxonomic group, involving the genes and their arrangement in the operon in which *pfs* is located. Here, the genes, *pfs*, *cbiB-cobD*, *btuF*, and *yeiH* are positioned according to their reading direction (Fig. 1B). In addition to the B_12_-dependent and -independent synthesis of methionine in the activated methyl cycle, the availability of B_12_ might also have an influence on this enzyme reaction.

Moreover, we observe significant differences in the B_12_ biosynthesis potential. Examining all available bacterial genomes, about 34% possess the complete B_12_ pathway, 51% lack B_12_ biosynthesis genes, and 15% are based on the genetic potential and possess the ability to scavenge cobamides. Among the *Gammaproteobacteria* genomes analyzed, the proportion identified as B_12_ prototrophs is 24%, lacking B_12_ biosynthesis is 51%, and those potentially capable of scavenging cobamides is 25%. In contrast, the fraction of *Vibrionales* genomes that are B_12_ prototrophs based on the same analysis is 14%, 29% lack B_12_ biosynthesis, and 57% are potentially cobamide scavengers.

### Growth characteristics of *V. campbellii*

**Fig 2:**
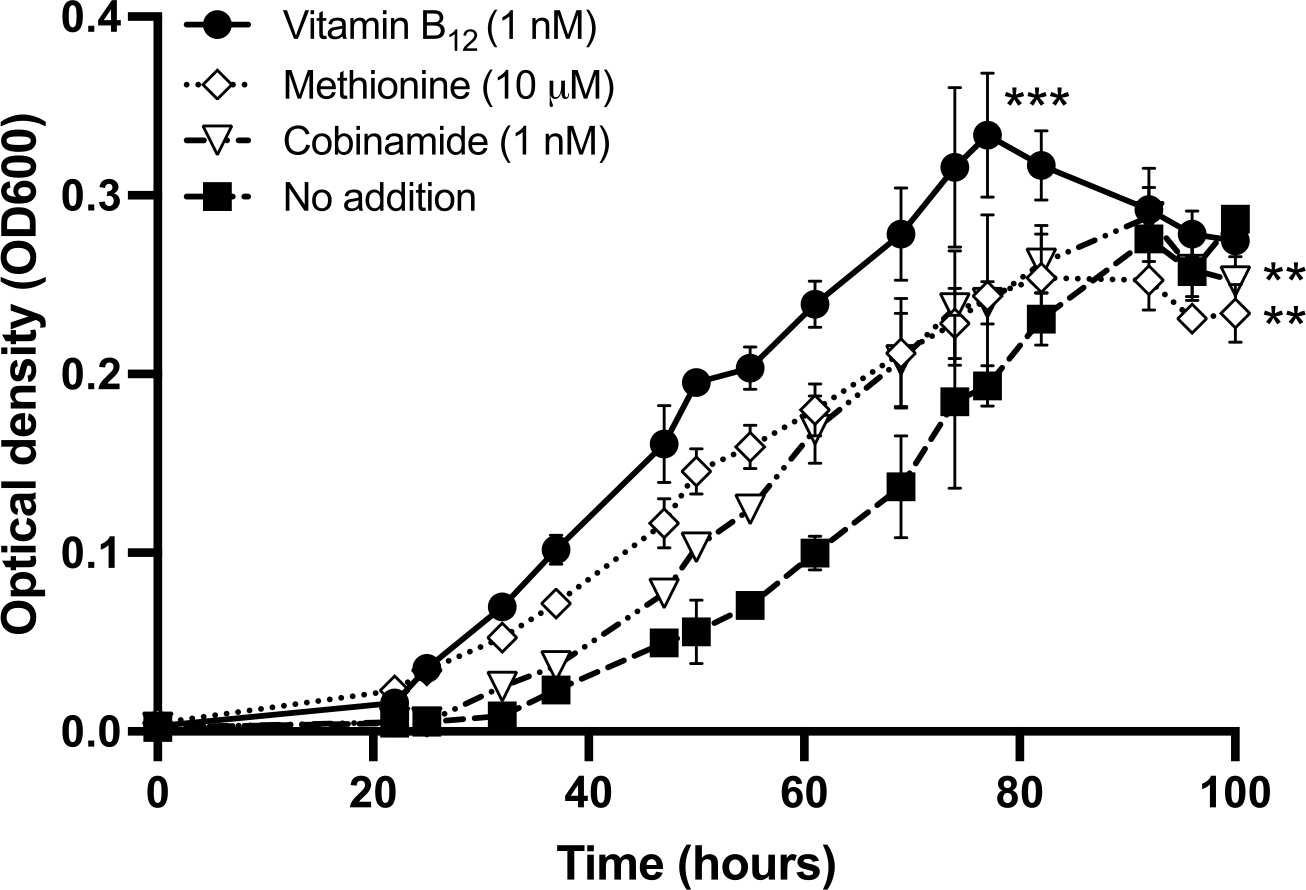
Growth curve of *V. campbellii* with supplementations of B_12_, methionine, and cobinamide. The growth of *V. campbellii* detected by optical density is shown over time (hours) with the addition of 1 nm B_12_ (white square), 10 µM methionine (black triangle), 1 nM cobinamide (white triangle), and without further supplementation (black circle). For each treatment, each point represents the mean ± SD of *n* = 3 independent biological replicates. Asterisks indicate significant differences (*t*-test, *P  <  0.05, **P  <  0.01, and ***P  <  0.001) compared to the negative control (no B_12_ added).

*V. campbellii* grows both with and without the availability of B_12_. When adding B_12_, however, *V. campbellii* reached its maximum optical density (OD) significantly faster with a slightly increased growth rate (Fig. 2; Table S2). When determining cell numbers in a follow-up experiment, it can also be observed that the growth rate is significantly increased by the addition of B_12_, and the cell numbers reach a similar peak (Fig. 4). Since OD measurements are a proxy for biomass concentrations and can vary due to culture conditions such as growth stage, culture stress, cell geometry, etc., the more accurate measurement method is the cell enumeration (47). The addition of cobinamide, the basic building block of all cobamides, which represents the corrin ring without an attached lower ligand, resulted in a slightly delayed growth of *V. campbellii* compared to the addition of B_12_ but an increased growth compared to cultivation without the addition of B_12_. By providing methionine only, *V. campbellii* reached its growth yield slightly faster compared to adding cobinamide but slightly slower than with the addition of B_12_ (Fig. 2).

### Detection of vitamin B_12_ and lower ligand

**Fig 3:**
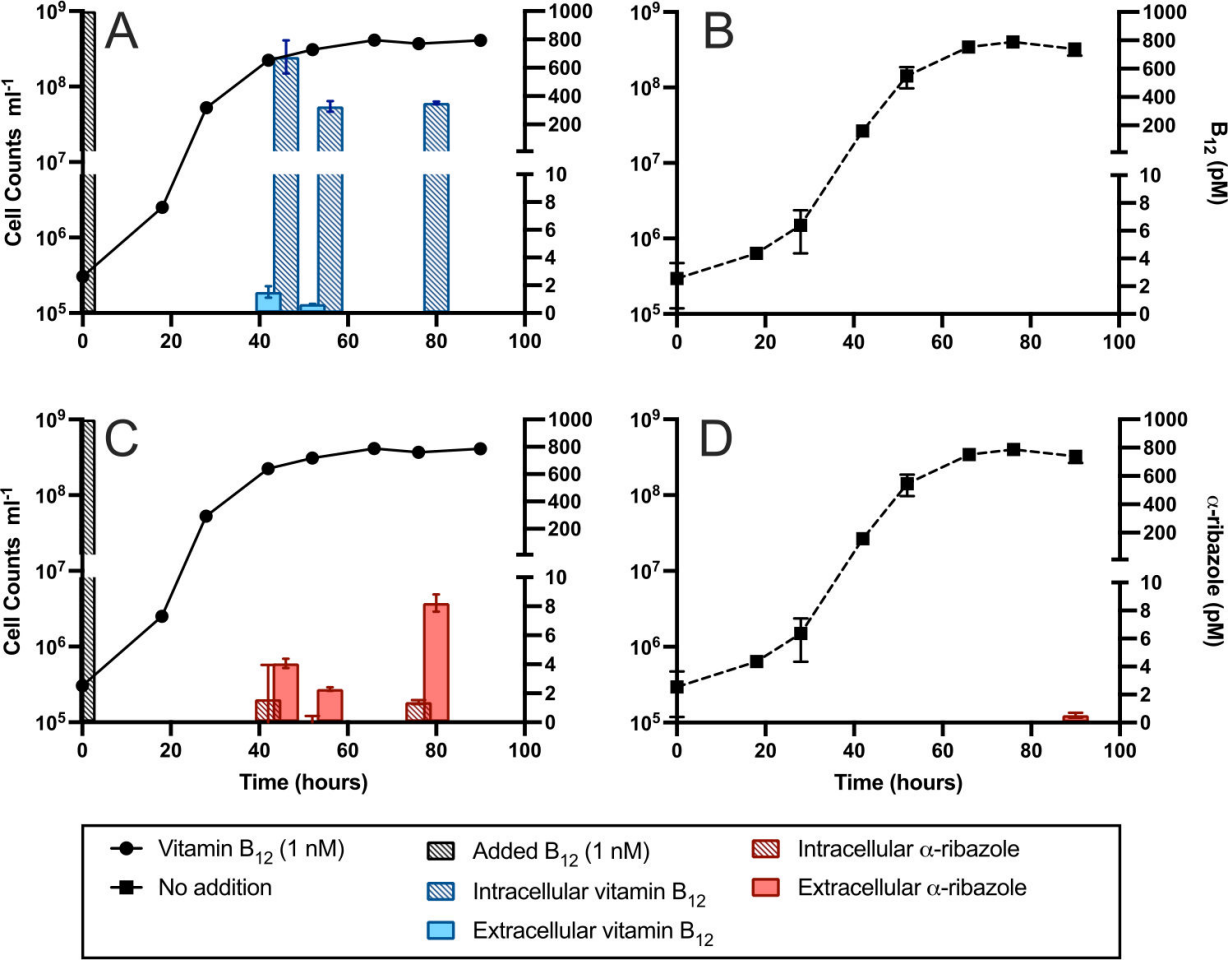
Extra- and intracellular B_12_ and lower ligand concentrations determined during growth of *V. campbellii*. The growth of *V. campbellii* (cell counts ml-1) over time (hours) with the addition of 1 nm B_12_ (black circle; **A and C**) and without any addition (white circle; **B and D**) is shown. For each treatment, each point represents the mean ± SD of *n* = 3 independent biological replicates. The bar plots represent the mean ± SD extracellular (blank) and intracellular (dashed) B_12_ (**A and B**) and lower ligand (**C and D**) concentrations.

*V. campbellii* was cultivated with the addition of 1 nM B_12_ and without. As expected, B_12_ was not detected in the culture without any artificial B_12_ addition (Fig. 3B). However, when adding 1 nM of B_12_, a miniscule concentration of B_12_ (1.5 ± 0.3 pM) was detected in the late exponential growth phase in the culture medium, whereas a concentration of 676.7 ± 95.1 pM B_12_ was detected intracellularly. In the early stationary growth phase, only 0.6 ± 0.02 pM B_12_ was measured in the culture medium, whereas 327.6 ± 30.6 pM B_12_ was measured intracellularly. Until the late stationary growth phase, the intracellularly detected B_12_ value remained stable, yet B_12_ was no longer detectable in the culture medium (Fig. 3).

Our LC-MS analysis also captured the lower ligand of B_12_, 5,6-dimethylbenzimidazole (DMB) and α-ribazole, which in addition to DMB also carries a ribose and is phosphorylated. DMB was not detected intracellularly in any of the *V. campbellii* samples nor in the medium. In contrast, α-ribazole was present both intracellularly and extracellularly in the medium with B_12_ supplementation, with an increasing trend and higher extracellular levels during the growth phase. The maximum was recorded in the late stationary phase, with a value of 8.2 ± 0.5 pM (Fig. 3C). In the *V. campbellii* culture without the addition of B_12_, α-ribazole was only detected in the late stationary phase in the medium with a value of 0.5 ± 0.1 pM (Fig. 3D).

### Autoinducer-2 detection

**Fig 4:**
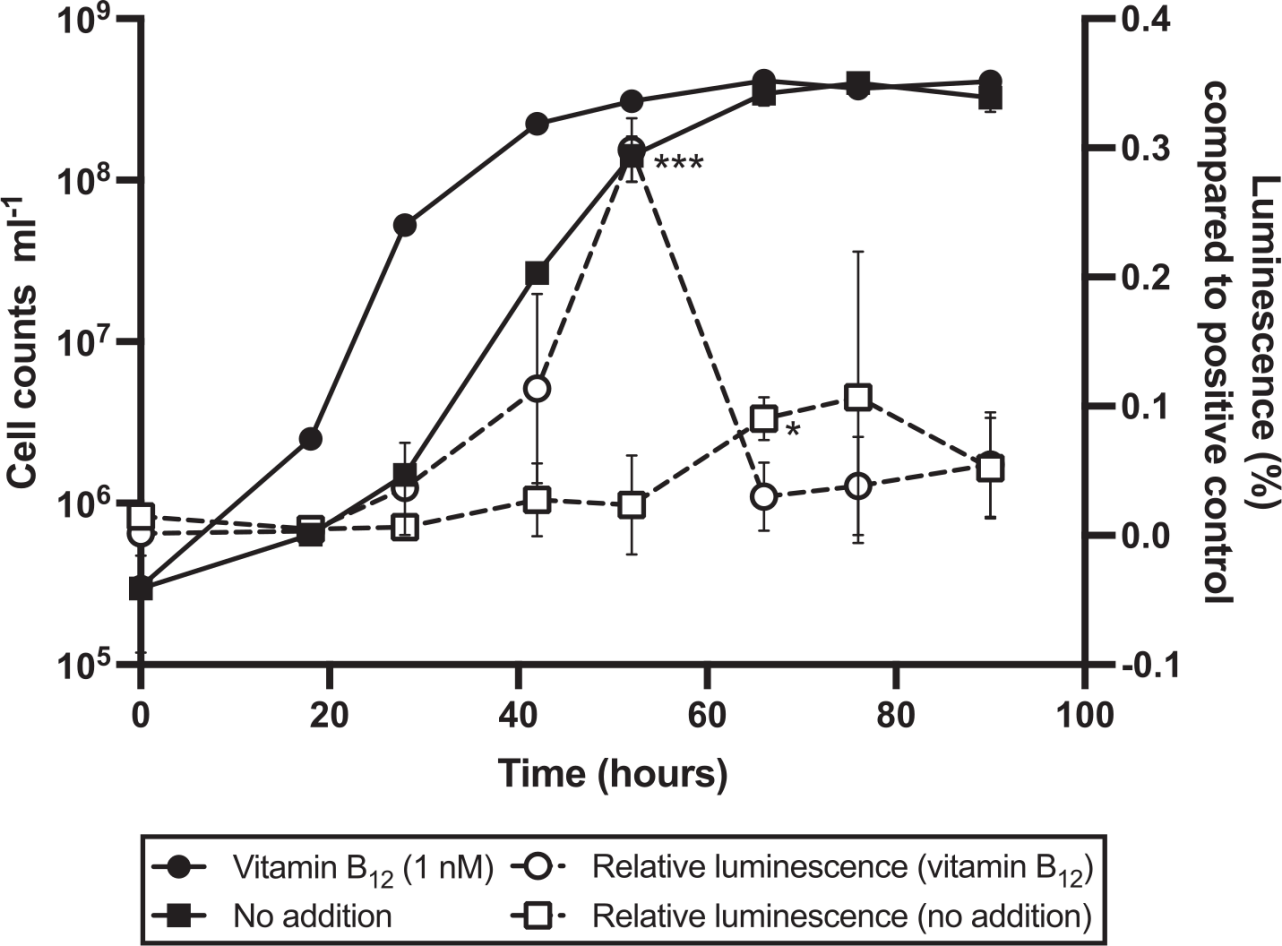
Extracellular autoinducer-2 concentrations detected during growth of *V. campbellii*. Shown are the growth of *V. campbellii* (cell counts per milliliter) over time (hours) with (black circle) and without (black square) the addition of B_12_. The detected AI-2 concentrations (luminescence percent compared to positive control) are shown over time (hours) with (white circle) and without (white square) the addition of B_12_. For each treatment, each point represents the mean ± SD of *n* = 3 independent biological replicates. Asterisks indicate significant differences (*t*-test, *P  <  0.05, **P  <  0.01, and ***P  <  0.001) compared to the negative control (no B_12_ added).

The AI-2 in the exometabolome of *V. campbellii* was measured using an established bioassay. When *V. campbellii* was cultivated with the addition of B_12_, we observed a strong increase in the extracellular level of AI-2 detected during the course of the exponential growth phase, with a clear maximum in the late exponential growth phase. During the stationary phase, the relative AI-2 value dropped back to the level at the time of the early exponential phase. A similar pattern of detected AI-2 levels over the course of the growth was observed in the *V. campbellii* culture without the addition of B_12_. Here, too, the value increased over the course of the growth experiment and reached its maximum in the early stationary phase. However, overall, the detected maximum of extracellular AI-2 of the culture with B_12_ addition was higher than that of the *V. campbellii* culture without B_12_ addition (Fig. 4).

### Effects of B_12_ availability on the transcriptome of *V. campbellii*

**Fig 5:**
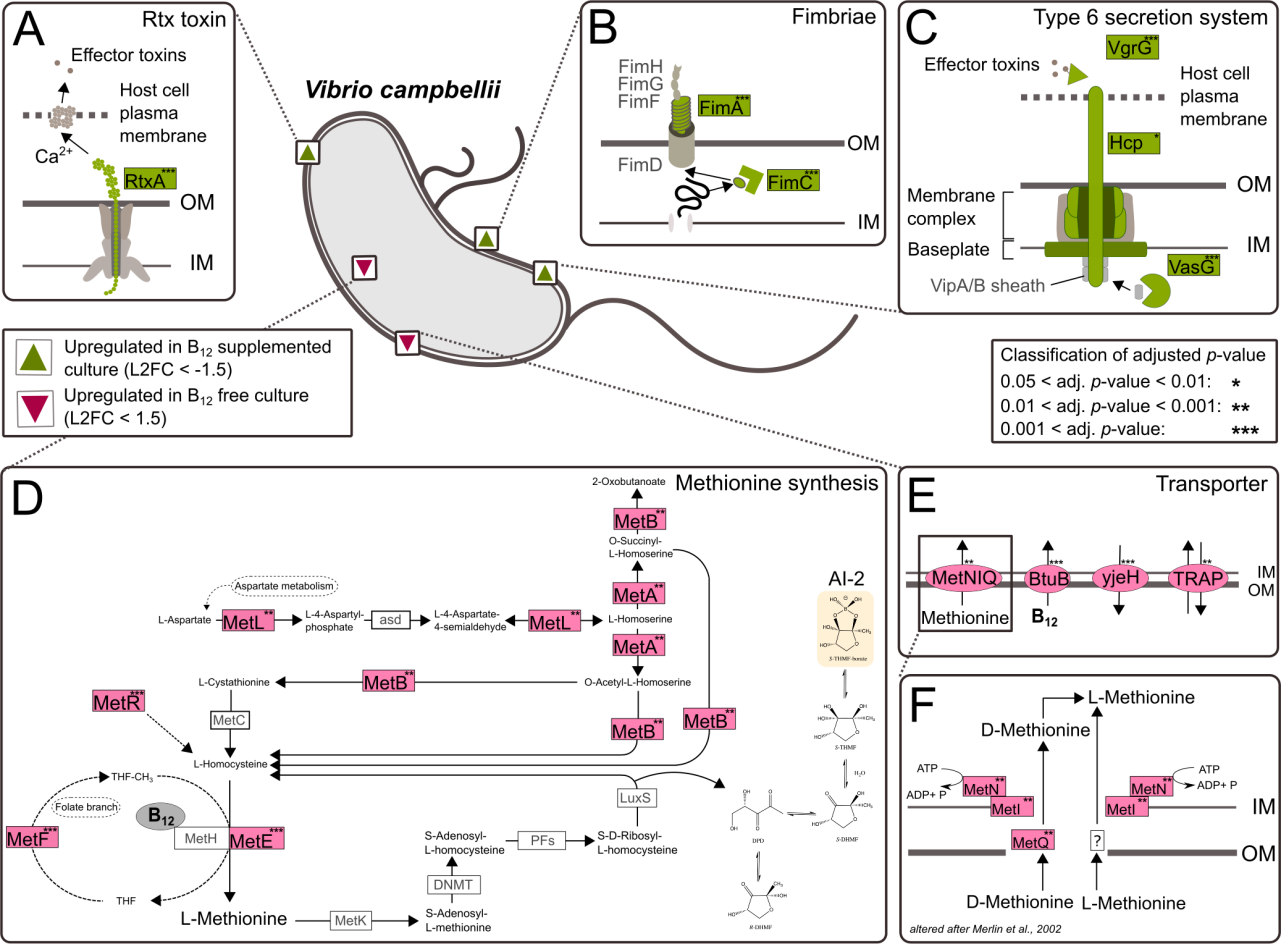
Illustration of a *V. campbelliii* cell with cellular mechanisms that are significantly differentially transcribed when B_12_ is supplied. Shown in this figure are genes significantly (adjusted *P*-value < 0.05) upregulated (green) or downregulated (red), with a log-fold change ±1.5 in B_12_-treated vs untreated *V. campbellii* cultures. Asterisks represent the adjusted *P*-value for respective genes (*P < 0.05, **P < 0.01, and ***P < 0.001). In A, the *rtxA* genes for the production of rtx-like toxin is shown. (B) Illustration of the fimbriae formation with the *fimA* and *fimC* genes significantly upregulated upon B_12_ addition. (C) Visualization of the T6SS with the significantly upregulated genes *vgrG*, *hcp*, *lip*, *dotU*, and *clpV* when B_12_ is provided. (D) Illustration of the methionine pathway and activated methyl cycle with the significantly upregulated genes *metA*, *metB*, *metL*, *metF*, *metH*, and *metE*. (E) Presentation of B_12_ outer membrane-binding protein BluB, L-methionine, and branched-chain amino acids exporter and the TRAP-transporter which were significantly upregulated at B_12_ deficiency and (**F)** with a distinct focus on the MetNIQ ABC methionine transporter.

B_12_ availability had a significant influence on the gene expression of *V. campbellii*. Differences identified and presented here derive from the comparison of the transcriptomes of *V. campbellii* cultures with and without B_12_ addition. In the absence of B_12_, expression of most methionine pathway genes was strongly and significantly upregulated, including *metL* (LOG2-FC = 2.2; padj = **), *metA* (LOG2-FC = 2.5; padj = **), and *metB* (LOG2-FC = 2.4; padj=**) that contribute decisively to the metabolic precursor synthesis of methionine, L-homocysteine. In addition, in the absence of B_12_, transcription of *metF* (LOG2-FC = 4.5; padj = ***) and the B_12_-independent methionine synthase *metE* (LOG2-FC = 7.3; padj = ***) were significantly and highly upregulated, while the B_12_-dependent *metH* (LOG2-FC = 1.4; padj=*) was also significantly but less pronounced upregulated. Furthermore, genes of a high-affinity methionine uptake system comprising *metN* (LOG2-FC = 2.1; padj = **), *metI* (LOG2-FC = 2.8; padj = **), and *metQ* (LOG2-FC = 2.0; padj = **), which belong to the ATP-binding cassettes, were significantly expressed when *V. campbellii* was lacking B_12_. Also, transcription of *yjeH* (LOG2-FC = 3.7; padj = ***), which enables the efflux of L-methionine (48), was highly upregulated. Furthermore, gene expression of *btuB* (LOG2-FC = 4.6; padj = ***) was strongly upregulated in the absence of B_12_, which aids the import of B_12_ into the periplasmic space by forming a complex with the inner membrane protein TonB (Fig. 5D through F; Data S2).

In turn, the provision of B_12_ to *V. campbellii* resulted also in distinct gene expression patterns. Particularly strong and significantly upregulated were T6SS genes. This includes the upregulation of all segments relevant for the pathogenicity of the T6SS. The *vgrG* (LOG2-FC = 2.4; padj = ***) gene, constituting the main part of the spike protein, *vasD* (LOG2-FC *=* 1.8; padj = **), *vasG* (LOG2-FC = 2.5; padj = ***), and *vasF* (LOG2-FC = 1.9; padj = ***), forming part of the membrane complex, and *impG* (LOG2-FC = 2.2; padj = ***), *impH* (LOG2-FC *= 1*.9; padj = ***), *impJ* (LOG2-FC = 2.1; padj = ***), and *vasJ* (LOG2-FC = 2.0; padj = **) the baseplate, as well as *hcp* (LOG2-FC = 2.0; padj = *) belonging to the sheath and tube. Transcription of *fimA* (LOG2-FC = 5.0; padj = ***) and *fimC* (LOG2-FC = 2.8; padj = ***), both involved in the formation of fimbriae, which is one virulence factor implicated in colonization, was also significantly upregulated when B_12_ was provided. A similar gene expression pattern was observed for *rtxA* (LOG2-FC = 2.5; padj = ***), which is critical for the formation of the RTX toxin (Fig. 5A through C; Data S2).

## DISCUSSION

### The facultative vitamin B_12_ consumer *V. campbellii*

*V. campbellii* grows both with and without the addition of B_12_, although growth is accelerated with the cofactor present. B_12_ auxotrophy of bacteria is usually manifested in the absence of the B_12_-independent methionine synthase. *V. campbellii* possesses both the B_12_-dependent (MetH) and -independent methionine synthase (MetE) and thus can synthesize methionine irrespective of B_12_ availability (Fig. 1). Therefore, *V. campbellii* is neither B_12_-auxotrophic nor B_12_-prototrophic; thus, we have categorized this strain as facultative vitamin B_12_ consumer. Similarly, both methionine synthases are encoded on most *Vibrionales* genomes analyzed here, rendering it likely for them to be facultative B_12_ consumers (Fig. 1A; Data S1), which in fact has been experimentally shown for some isolates (24).

Encoding both *metH* and *metE* may confer an ecological advantage for *Vibrionales* and other bacteria (49), as B_12_ is a valuable and rare commodity in many ecosystems (6, 46, 50, 51). The ability to synthesize methionine *de novo* under B_12_-limited conditions may provide an advantage over B_12_-auxotrophic strains. The simultaneous encoding of *metH* by many *Vibrionales* spp. could be explained by the high methionine synthesis efficiency in the presence of B_12_ (13, 14). However, our observation of accelerated growth with B_12_ addition cannot be fully attributed to methionine synthases, as the provision of methionine does not equally increase growth of *V. campbellii* (Fig. 2). Consequently, intracellular processes beyond methionine synthesis are probably affected by B_12_ availability. Furthermore, encoded genes for lower ligand synthesis and nucleotide loop assembly in *V. campbellii*, along with the affected growth upon Cbi addition, indicate the cofactor’s relevance and suggest that *V. campbellii* possesses the ability to remodel unsuitable cobamides. Our observation of a rapid intracellular B_12_ decline after complete consumption of dissolved B_12_ supports our assumption and indeed the ability of *V. cholerae*, which is related to *V. campbellii*, to convert unsuitable cobamides such as pseudocobalamin has been demonstrated (24).

### Impact of B_12_ availability on *V. campbellii*

With B_12_ absent, the growth rate of *V. campbellii* was reduced when determined by optical density, and distinct cellular processes were differentially expressed. Decelerated growth may reflect, in part, a lower methionine synthesis efficiency utilizing the B_12_-independent methionine synthase (13, 14). Transcription of genes for methionine synthesis and transport (*metA*, *metB*, and *metL*) and the regulator *metR*, a *lysR* family regulatory protein, known to positively regulate *metA*, *metF*, *metE*, and *metH* (Fig. 5D), was highly upregulated in the absence of B_12_ (52).

Contrary to previous studies suggesting B_12_-dependent methionine synthase is only expressed in the presence of B_12_, we demonstrated here that both methionine synthase genes, *metE* and *metH*, are upregulated in the absence of B_12_ (53, 54). This finding suggests that *metE* and *metH* are not mutually regulated in *V. campbellii* but rather can be simultaneously expressed at high levels, enabling rapid adaptions to accessible B_12_. Also, *btuB*, the B_12_-binding outer membrane protein, was highly upregulated under B_12_ deficiency, indicating a B_12_ requirement during *metH* expression (Fig. 5D and E).

In contrast, B_12_ supply accelerated growth, extracellular AI-2 levels, and virulence factor gene expression in *V. campbellii*, such as toxin synthesis, fimbria formation, and the activation of the T6SS. B_12_ availability has been previously linked to the expression of virulence factor genes. Recent studies show that increased B_12_ exposure can alter the gut microbiome, leading to bacterial pathogenicity (55), and additionally, reduced B_12_ availability, caused by *Bacteroides thetaiotaomicron*, lowers Shiga toxin production in *Escherichia coli* (56). Furthermore, many clinical studies have shown that increased B_12_ availability is associated with the development of acne (57–61), probably reflecting the impact of B_12_ on the skin microbiota (62). While all these studies point to a link between enhanced B_12_ availability and bacterial infectious diseases, the direct mechanism of action has remained uncertain.

In our study, *rtxA* and a gene for a Ca^2+^-binding RTX toxin-like protein (COG2931) were highly transcribed in the presence of B_12_. Both genes are related to the RTX toxin family, a group of associated exotoxins produced by a variety of pathogenic Gram-negative bacteria (63). The *rtxA* gene encodes a multifunctional autoprocessing repeat-in-toxin (MARTX toxin) that belongs to a heterogeneous group of toxins found in *Vibrio* spp. and other Gram-negative bacteria (64). Secreted by the type-1 secretion system, RTX has a cytotoxic effect on various host cells and is closely associated with the virulence of several *Vibrio* species (64–66) (Fig. 5A). Also, gene transcription of fimbriae (*fimA* and *fimC*), key virulence factors in many *Vibrio* species that enable bacterial attachment to surfaces and are essential for biofilm formation and host cell interaction, was highly upregulated (67). In *E. coli*, for example, the type 1 fimbriae is decisive for the host colonization and can therefore influence pathogenicity (68) (Fig. 5B). Furthermore, gene expression of *vgrG*, *hcp*, *lip*, *dotU*, and *clpV* was upregulated, accounting for important components from spike to tube of the T6SS, a molecular machinery commonly used by Gram-negative bacteria to deliver their toxins through the cell envelope into neighboring cells (69) (Fig. 5C).

### Possible link between vitamin B_12_ and autoinducer-2 synthesis

The efficiency of methionine synthesis efficiency strongly depends upon the encoded methionine synthase, as the B_12_-dependent methionine synthase (MetH) has a much higher reaction rate compared to the B_12_-independent methionine synthase (MetE) (13, 14). Certain bacteria, including most *Vibrionales* strains, possess both MetH and MetE, and most *Vibrionales* strains cannot synthesize B_12_ *de novo* (Fig. 1A; Data S1); thus, its availability is likely to affect methionine synthesis. Methionine is essential for various vital cellular processes and sets the basis for L-adenosyl homocysteine synthesis in the activated methyl cycle, which in turn is the precursor for various AHLs, including AI-2. While AI-2 is not essential for bacterial survival, it functions as a quorum-sensing compound, regulating intracellular processes and enabling interspecies signaling.

We speculate if less methionine is synthesized upon B_12_ deficiency, methionine is more likely to be directed to essential cellular functions and therefore affects AI-2 synthesis. The enzymes PFs and LuxS, in the two-step reaction of the activated methyl cycle, mediate the synthesis of AI-2 from S-adenosyl-L-homocysteine (Fig. 1). In *Salmonella enterica*, *pfs* and *btuF* are likely co-transcribed in the same operon since the insertion of an antibiotic resistance gene into *pfs* resulted in the inhibition of B_12_ uptake (70–72). The cofactor enters the cell through the outer membrane via the receptor protein BtuB by the aid of TonB. In the periplasm, B_12_ is scavenged by the BtuF protein and then further transferred to the inner membrane complex BtuCD into the cytoplasm (72–74). Thus, already Beeston et al. (2002) speculated (71) that B_12_ availability was directly related to AI-2 synthesis. However, to our knowledge, this reasonable conclusion was never pursued in detail or investigated for other pathogenic bacteria outside the order *Enterobacterales*. Here, we have examined the operon encoding *pfs* in various *Vibrionales* genomes. Alongside the B_12_-binding gene *btuF*, we also identified *cobD* in most *Vibrionales* genomes, which is directly related to B_12_ acquisition. This gene encodes for an essential enzyme for cobamide or cobinamide salvaging. In our analysis, 57% of *Vibrionales* genomes constitutes the genetic potential to salvage cobamides or cobinamides (Fig. 1C; Data S1). Overall, we can note that the vast majority of analyzed *Vibrionales* strains encode *pfs*, *cobD*/*cobB*, *btuF*, and *yeiH* in one operon (Fig. S1). Based on the links and findings presented here, we hypothesize that in *V. campbellii*, which encodes both the B_12_-dependent and -independent methionine synthase, the availability of B_12_ has a direct effect on the synthesis of AI-2. In fact, we determined a higher relative AI-2 value in cultures with B_12_.

This possible link between B_12_ availability and AI-2 production may explain our observation of a multitude of highly expressed virulence factor genes in *V. campbellii* when we provided B_12_. In fact, there is ample evidence that AI-2 significantly affects the regulation of virulence factors of a variety of gammaproteobacterial strains, including those being upregulated in our study (75–77). For example, leukotoxin, a member of the RTX family of toxins, is regulated in an AI-2-dependent manner (78). Similarly, there are indications that the biosynthesis of fimbriae in certain bacteria is mediated via AI-2 (75). Also, the transcription of T6SS genes can be regulated by quorum-sensing molecules, such as AI-2 (16, 17, 22, 79). It is assumed that AI-2 is sensed by the cell surface receptor LuxPQ, which at high concentrations leads to the activation of the receptor LuxO, resulting in the synthesis of LuxR. This in turn triggers the synthesis of HapR, a quorum-sensing regulator, which itself triggers virulence factors, including the T6SS of *V. campbellii* (16, 76).

### Conclusion

Multiple studies have shown that increased B_12_ availability can enhance the virulence of individual pathogenic bacteria or result in an increase of bacterial infectious diseases (55–62). Our study confirms that different virulence factors, such as toxin synthesis, fimbria formation, and the activation of the T6SS, of a specific bacterial isolate, *V. campbellii*, are significantly transcribed when B_12_ is available. In the following, we provide a possible explanation for this observation.

*V. campbellii*, like most *Vibrio* spp. incapable of synthesizing B_12_ *de novo*, encodes both the B_12_-independent (MetE) and B_12_-dependent methionine synthase (MetH), enabling growth with and without B_12_. However, the availability of B_12_ leads to accelerated growth of *V. campbellii*, which is likely in part associated to an enhanced methionine synthesis. The basic building block of quorum-sensing molecules, including AI-2, which significantly regulates virulence factors in *Vibrio* spp., is S-adenosyl L-homocysteine, which itself requires methionine as a metabolic precursor. In our study, we show that in the absence of B_12_, genes of methionine synthesis are significantly upregulated, and extracellular AI-2 availability is significantly lower. We anticipate that increased synthesis of methionine upon availability of B_12_ triggers a cascade effect, whereby more S-adenosyl L-homocysteine is synthesized and consequently also increased AI-2 levels. Our assumption of a direct link between AI-2 synthesis and B_12_ acquisition is also supported by the observation that *pfs*, which initiates the two-step synthesis of AI-2, has two crucial genes encoded on the same operon, relevant for the uptake and bioavailability of B_12_. Since our conclusion of the relationship between B_12_ availability, synthesis of methionine and thus AI-2 as well as virulence factor regulation of *V. campbellii* is based solely on successive independent experiments and analyses, it would be of great value if this relationship is also demonstrated by means of knockout mutation experiments.

The evolutionary origins of such a relationship between B_12_ and AI-2 remain speculative. Since only few prokaryotes produce or share B_12_ (3, 6), increased availability may signal high microbial activity in nature. Virulence factors of pathogenic bacteria are often regulated by quorum sensing, which depends strongly on bacterial cell density (80). *V. campbellii*, which is pathogenic to various aquatic organisms (81, 82), grows faster with B_12_, and its availability upregulates virulence factor gene expression, potentially resulting in the death of other microorganisms. This then presumably leads to increased B_12_ and organic compound availability, thereby promoting rapid growth.

## Data Availability

Transcriptomic sequences generated in this study were deposited in European Nucleotide Archive (ENA) at EMBL-EBI with the accession number PRJEB66273.

## SUPPLEMENTAL MATERIAL

Data S1 aem.01422-24-s0001.xlsx Listed are 3716 bacterial genomes, publicly available on https://img.jgi.doe.gov/, that possess at least 54 of the 55 surveyed housekeeping genes.

Data S2 aem.01422-24-s0002.xlsx Transcriptional gene regulation of *V. campbellii* culture when supplemented with and without the addition of B_12_ (1 nM).

Fig. S1 aem.01422-24-s0003.pdf Operon encoding pfs in the genome of *V. campbellii*, which is the first gene of the two-step AI-2 synthesis.

Supplemental legends aem.01422-24-s0004.docx Legends for supplemental material.

Table S1 aem.01422-24-s0005.docx Extra- and intracellular B_12_ (cyanocobalamin, adenosylcobalamin, methylcobalamin, hydroxycobalamin) and activated lower ligand (α-ribazole) recovery by LC-MS in *V. campbellii* cultures.

Table S2 aem.01422-24-s0006.docx Growth time, yield, and rate of *V. campbellii* determined by optical density when growing in mono-culture with (1 nM) and without B_12_ supplementation or the addition of methionine (10 µM) or cobinamide (1 nM).

ASM does not own the copyrights to Supplemental Material that may be linked to, or accessed through, an article. The authors have granted ASM a non-exclusive, world-wide license to publish the Supplemental Material files. Please contact the corresponding author directly for reuse.

## References

[1] Roth JR, Lawrence JG, Bobik TA. 1996. Cobalamin (coenzyme B_12_): synthesis and biological significance. Annu Rev Microbiol 50:137–181. doi:10.1146/annurev.micro.50.1.137 8905078

[2] Shelton AN, Seth EC, Mok KC, Han AW, Jackson SN, Haft DR, Taga ME. 2019. Uneven distribution of cobamide biosynthesis and dependence in bacteria predicted by comparative genomics. ISME J 13:789–804. doi:10.1038/s41396-018-0304-9 30429574 PMC6461909

[3] Sañudo-Wilhelmy SA, Gómez-Consarnau L, Suffridge C, Webb EA. 2014. The role of B vitamins in marine biogeochemistry. Ann Rev Mar Sci 6:339–367. doi:10.1146/annurev-marine-120710-100912 24050603

[4] Swaney MH, Sandstrom S, Kalan LR. 2022. Cobamide sharing is predicted in the human skin microbiome. mSystems 7:e0067722. doi:10.1128/msystems.00677-22 35968974 PMC9600381

[5] Lu X, Heal KR, Ingalls AE, Doxey AC, Neufeld JD. 2020. Metagenomic and chemical characterization of soil cobalamin production. ISME J 14:53–66. doi:10.1038/s41396-019-0502-0 31492962 PMC6908642

[6] Sultana S, Bruns S, Wilkes H, Simon M, Wienhausen G. 2023. Vitamin B_12_ is not shared by all marine prototrophic bacteria with their environment. ISME J 17:836–845. doi:10.1038/s41396-023-01391-3 36914732 PMC10203341

[7] Wienhausen G, Moraru C, Bruns S, Tran DQ, Sultana S, Wilkes H, Dlugosch L, Azam F, Simon M. 2024. Ligand cross-feeding resolves bacterial vitamin B_12_ auxotrophies. Nature New Biol 629:886–892. doi:10.1038/s41586-024-07396-y 38720071

[8] Payne KA, Quezada CP, Fisher K, Dunstan MS, Collins FA, Sjuts H, Levy C, Hay S, Rigby SE, Leys D. 2015. Reductive dehalogenase structure suggests a mechanism for B_12_-dependent dehalogenation. Nature New Biol 517:513–516. doi:10.1038/nature13901 PMC4968649 25327251

[9] Mandal M, Breaker RR. 2004. Gene regulation by riboswitches. Nat Rev Mol Cell Biol 5:451–463. doi:10.1038/nrm1403 15173824

[10] Johnson Jr JE, Reyes FE, Polaski JT, Batey RT. 2012. B_12_ cofactors directly stabilize an mRNA regulatory switch. Nature New Biol 492:133–137. doi:10.1038/nature11607 PMC3518761 23064232

[11] Drennan CL, Matthews RG, Ludwig ML. 1994. Cobalamin-dependent methionine synthase: the structure of a methylcobalamin-binding fragment and implications for other B_12_-dependent enzymes. Curr Opin Struct Biol 4:919–929. doi:10.1016/0959-440x(94)90275-5 7712296

[12] Ferla MP, Patrick WMY. 2014. Bacterial methionine biosynthesis. Microbiology (Reading) 160:1571–1584. doi:10.1099/mic.0.077826-0 24939187

[13] Figge RM. 2007. Methionine biosynthesis in *Escherichia coli* and *Corynebacterium glutamicu*m, p 163–193. In Wendisch VF (ed), Amino acid biosynthesis ~ pathways, regulation and metabolic engineering. Springer, Berlin, Heidelberg.

[14] Drummond JT, Matthews RG. 1993. Cobalamin-dependent and cobalamin-independent methionine synthases in Escherichia coli: two solutions to the same chemical problem. Adv Exp Med Biol 338:687–692. doi:10.1007/978-1-4615-2960-6_142 8304207

[15] Kumar S, Kumar CB, Rajendran V, Abishaw N, Anand PSS, Kannapan S, Nagaleekar VK, Vijayan KK, Alavandi SV. 2021. Delineating virulence of Vibrio campbellii: a predominant luminescent bacterial pathogen in Indian shrimp hatcheries. Sci Rep 11:15831. doi:10.1038/s41598-021-94961-4 34349168 PMC8339124

[16] Noor Nm, Defoirdt T, Alipiah N, Karim M, Daud H, Natrah I. 2019. Quorum sensing is required for full virulence of Vibrio campbellii towards tiger grouper (Epinephelus fuscoguttatus) larvae. J Fish Dis 42:489–495. doi:10.1111/jfd.12946 30742313

[17] Han Y, Pan J, Huang Y, Cheng Q, Liu P, Diao B, Li J, Kan B, Liang W. 2022. VfqI-VfqR quorum sensing circuit modulates type VI secretion system VflT6SS2 in Vibrio fluvialis. Biochem Biophys Rep 31:101282. doi:10.1016/j.bbrep.2022.101282 35669988 PMC9166416

[18] Miller MB, Skorupski K, Lenz DH, Taylor RK, Bassler BL. 2002. Parallel quorum sensing systems converge to regulate virulence in Vibrio cholerae*.* Cell 110:303–314. doi:10.1016/s0092-8674(02)00829-2 12176318

[19] Zhu J, Miller MB, Vance RE, Dziejman M, Bassler BL, Mekalanos JJ. 2002. Quorum-sensing regulators control virulence gene expression in Vibrio cholerae. Proc Natl Acad Sci U S A 99:3129–3134. doi:10.1073/pnas.052694299 11854465 PMC122484

[20] Vendeville A, Winzer K, Heurlier K, Tang CM, Hardie KR. 2005. Making “sense” of metabolism: autoinducer-2, LUXS and pathogenic bacteria. Nat Rev Microbiol 3:383–396. doi:10.1038/nrmicro1146 15864263

[21] Metzger LC, Matthey N, Stoudmann C, Collas EJ, Blokesch M. 2019. Ecological implications of gene regulation by TfoX and TfoY among diverse Vibrio species. Environ Microbiol 21:2231–2247. doi:10.1111/1462-2920.14562 30761714 PMC6618264

[22] Leung KY, Siame BA, Snowball H, Mok Y-K. 2011. Type VI secretion regulation: crosstalk and intracellular communication. Curr Opin Microbiol 14:9–15. doi:10.1016/j.mib.2010.09.017 20971679

[23] Ishikawa T, Rompikuntal PK, Lindmark B, Milton DL, Wai SN. 2009. Quorum sensing regulation of the two hcp alleles in Vibrio cholerae O1 strains. PLoS One 4:e6734. doi:10.1371/journal.pone.0006734 19701456 PMC2726435

[24] Agarwal S, Dey S, Ghosh B, Biswas M, Dasgupta J. 2019. Mechanistic basis of vitamin B_12_ and cobinamide salvaging by the Vibrio species. Biochim Biophys Acta Proteins Proteom 1867:140–151. doi:10.1016/j.bbapap.2018.11.004 30463026

[25] Ma AT, Beld J, Brahamsha B. 2017. An amoebal grazer of cyanobacteria requires cobalamin produced by heterotrophic bacteria. Appl Environ Microbiol 83:e00035-17. doi:10.1128/AEM.00035-17 28283521 PMC5411508

[26] Ma AT, Tyrell B, Beld J. 2020. Specificity of cobamide remodeling, uptake and utilization in Vibrio cholerae*.* Mol Microbiol 113:89–102. doi:10.1111/mmi.14402 31609521

[27] Bodor A, Elxnat B, Thiel V, Schulz S, Wagner-Döbler I. 2008. Potential for luxS related signalling in marine bacteria and production of autoinducer-2 in the genus Shewanella. BMC Microbiol 8:13. doi:10.1186/1471-2180-8-13 18215278 PMC2233627

[28] Taga ME. 2006. Methods for analysis of bacterial autoinducer‐2 production. CP Microbiology 00:1. doi:10.1002/9780471729259.mc01c01s00 18770547

[29] Giebel H-A, Wolterink M, Brinkhoff T, Simon M. 2019. Complementary energy acquisition via aerobic anoxygenic photosynthesis and carbon monoxide oxidation by Planktomarina temperata of the Roseobacter group. FEMS Microbiol Ecol 95:fiz050. doi:10.1093/femsec/fiz050 31055603

[30] Blair WM, Doucette GJ. 2013. The Vibrio harveyi bioassay used routinely to detect AI-2 quorum sensing inhibition is confounded by inconsistent normalization across marine matrices. J Microbiol Methods 92:250–252. doi:10.1016/j.mimet.2012.12.023 23305926

[31] Markowitz VM, Chen I-MA, Palaniappan K, Chu K, Szeto E, Grechkin Y, Ratner A, Jacob B, Huang J, Williams P, Huntemann M, Anderson I, Mavromatis K, Ivanova NN, Kyrpides NC. 2012. IMG: the Integrated Microbial Genomes database and comparative analysis system. Nucleic Acids Res 40:D115–D122. doi:10.1093/nar/gkr1044 22194640 PMC3245086

[32] Raes J, Korbel JO, Lercher MJ, von Mering C, Bork P. 2007. Prediction of effective genome size in metagenomic samples. Genome Biol 8:R10. doi:10.1186/gb-2007-8-1-r10 17224063 PMC1839125

[33] Brown CT, Hug LA, Thomas BC, Sharon I, Castelle CJ, Singh A, Wilkins MJ, Wrighton KC, Williams KH, Banfield JF. 2015. Unusual biology across a group comprising more than 15% of domain bacteria. Nature New Biol 523:208–211. doi:10.1038/nature14486 26083755

[34] Bolger AM, Lohse M, Usadel B. 2014. Trimmomatic: a flexible trimmer for Illumina sequence data. Bioinformatics 30:2114–2120. doi:10.1093/bioinformatics/btu170 24695404 PMC4103590

[35] Kopylova E, Noé L, Touzet H. 2012. SortMeRNA: fast and accurate filtering of ribosomal RNAs in metatranscriptomic data. Bioinformatics 28:3211–3217. doi:10.1093/bioinformatics/bts611 23071270

[36] Quast C, Pruesse E, Yilmaz P, Gerken J, Schweer T, Yarza P, Peplies J, Glöckner FO. 2013. The SILVA ribosomal RNA gene database project: improved data processing and web-based tools. Nucleic Acids Res 41:D590–D596. doi:10.1093/nar/gks1219 23193283 PMC3531112

[37] Griffiths-Jones S, Bateman A, Marshall M, Khanna A, Eddy SR. 2003. Rfam: an RNA family database. Nucleic Acids Res 31:439–441. doi:10.1093/nar/gkg006 12520045 PMC165453

[38] Langmead B, Salzberg SL. 2012. Fast gapped-read alignment with Bowtie 2. Nat Methods 9:357–359. doi:10.1038/nmeth.1923 22388286 PMC3322381

[39] Love MI, Huber W, Anders S. 2014. Moderated estimation of fold change and dispersion for RNA-seq data with DESeq2. Genome Biol 15:550. doi:10.1186/s13059-014-0550-8 25516281 PMC4302049

[40] Benjamini Y, Hochberg Y. 1995. Controlling the false discovery rate: a practical and powerful approach to multiple testing. J R Stat Soc Ser B 57:289–300. doi:10.1111/j.2517-6161.1995.tb02031.x

[41] Diepenbroek M, Glöckner FO, Grobe P, Güntsch A, Huber R, König-Ries B, Kostadinov I, Nieschulze J, Seeger B, Tolksdorf R, Triebel D. 2014. Towards an integrated biodiversity and ecological research data management and archiving platform: the German federation for the curation of biological data (GFBio). Gesellschaft für Informatik e.V. Available from: https://dl.gi.de/items/618c1a92-fff2-423e-8c60-a0d6a65fe04f. Retrieved 1 Sep 2023.

[42] Yilmaz P, Kottmann R, Field D, Knight R, Cole JR, Amaral-Zettler L, Gilbert JA, Karsch-Mizrachi I, Johnston A, Cochrane G, et al.. 2011. Minimum information about a marker gene sequence (MIMARKS) and minimum information about any (x) sequence (MIxS) specifications. Nat Biotechnol 29:415–420. doi:10.1038/nbt.1823 21552244 PMC3367316

[43] Bruns S, Wienhausen G, Scholz-Böttcher B, Wilkes H. 2022. Simultaneous quantification of all B vitamins and selected biosynthetic precursors in seawater and bacteria by means of different mass spectrometric approaches. Anal Bioanal Chem 414:7839–7854. doi:10.1007/s00216-022-04317-8 36195729 PMC9568461

[44] Bruns S, Wienhausen G, Scholz-Böttcher B, Heyen S, Wilkes H. 2023. Method development and quantification of all B vitamins and selected biosynthetic precursors in winter and spring samples from the North Sea and de novo synthesized by Vibrio campbellii*.* Mar Chem 256:104300. doi:10.1016/j.marchem.2023.104300

[45] Cakić N, Kopke B, Rabus R, Wilkes H. 2021. Suspect screening and targeted analysis of acyl coenzyme a thioesters in bacterial cultures using a high-resolution tribrid mass spectrometer. Anal Bioanal Chem 413:3599–3610. doi:10.1007/s00216-021-03318-3 33881564 PMC8141488

[46] Wienhausen G, Dlugosch L, Jarling R, Wilkes H, Giebel H-A, Simon M. 2022. Availability of vitamin B_12_ and its lower ligand intermediate α-ribazole impact prokaryotic and protist communities in oceanic systems. ISME J 16:2002–2014. doi:10.1038/s41396-022-01250-7 35585186 PMC9296465

[47] Myers JA, Curtis BS, Curtis WR. 2013. Improving accuracy of cell and chromophore concentration measurements using optical density. BMC Biophys 6:4. doi:10.1186/2046-1682-6-4 24499615 PMC3663833

[48] Liu Q, Liang Y, Zhang Y, Shang X, Liu S, Wen J, Wen T. 2015. YjeH is a novel exporter of l-methionine and branched-chain amino acids in Escherichia coli*.* Appl Environ Microbiol 81:7753–7766. doi:10.1128/AEM.02242-15 26319875 PMC4616930

[49] Mars Brisbin M, Schofield A, McIlvin MR, Krinos AI, Alexander H, Saito MA. 2023. Vitamin B_12_ conveys a protective advantage to phycosphere-associated bacteria at high temperatures. ISME Commun 3:88. doi:10.1038/s43705-023-00298-6 37626172 PMC10457287

[50] Koch F, Marcoval MA, Panzeca C, Bruland KW, Sañudo-Wilhelmy SA, Gobler CJ. 2011. The effect of vitamin B _12_ on phytoplankton growth and community structure in the Gulf of Alaska. Limnol Oceanogr 56:1023–1034. doi:10.4319/lo.2011.56.3.1023

[51] Bertrand EM, Saito MA, Rose JM, Riesselman CR, Lohan MC, Noble AE, Lee PA, DiTullio GR. 2007. Vitamin B _12_ and iron colimitation of phytoplankton growth in the Ross Sea. Limnol Oceanogr 52:1079–1093. doi:10.4319/lo.2007.52.3.1079

[52] Chatterjee J, Miyamoto CM, Zouzoulas A, Lang BF, Skouris N, Meighen EA. 2002. MetR and CRP bind to the Vibrio harveyi lux promoters and regulate luminescence. Mol Microbiol 46:101–111. doi:10.1046/j.1365-2958.2002.03128.x 12366834

[53] Hondorp ER, Matthews RG. 2004. Oxidative stress inactivates cobalamin-independent methionine synthase (MetE) in Escherichia coli*.* PLoS Biol 2:e336. doi:10.1371/journal.pbio.0020336 15502870 PMC521173

[54] Urbanowski ML, Stauffer GV. 1989. Role of homocysteine in metR-mediated activation of the metE and metH genes in Salmonella typhimurium and Escherichia coli. J Bacteriol 171:3277–3281. doi:10.1128/jb.171.6.3277-3281.1989 2656646 PMC210046

[55] Forgie AJ, Pepin DM, Ju T, Tollenaar S, Sergi CM, Gruenheid S, Willing BP. 2023. Over supplementation with vitamin B_12_ alters microbe-host interactions in the gut leading to accelerated Citrobacter rodentium colonization and pathogenesis in mice. Microbiome 11:21. doi:10.1186/s40168-023-01461-w 36737826 PMC9896722

[56] Cordonnier C, Le Bihan G, Emond-Rheault J-G, Garrivier A, Harel J, Jubelin G. 2016. Vitamin B_12_ uptake by the gut commensal bacteria bacteroides thetaiotaomicron limits the production of shiga toxin by enterohemorrhagic Escherichia coli. Toxins (Basel) 8:14. doi:10.3390/toxins8010014 26742075 PMC4728536

[57] Veraldi S, Benardon S, Diani M, Barbareschi M. 2018. Acneiform eruptions caused by vitamin B_12_: a report of five cases and review of the literature. J Cosmet Dermatol 17:112–115. doi:10.1111/jocd.12360 28594082

[58] Balta I, Ozuguz P. 2014. Vitamin B_12_-induced acneiform eruption. Cutan Ocul Toxicol 33:94–95. doi:10.3109/15569527.2013.808657 23815241

[59] Puissant A, Monfort J, Vanbremeersch F. 1969. Acne caused by vitamin B-12. Gaz Med Fr 76:4535–4539.

[60] Zamil DH, Perez-Sanchez A, Katta R. 2020. Acne related to dietary supplements. Dermatol Online J 26:13030/qt9rp7t2p2. 32941710

[61] Arora MK, Seth S, Dayal S. 2012. Homocysteine, folic acid and vitamin B_12_ levels in females with severe acne vulgaris. Clin Chem Lab Med 50:2061–2063. doi:10.1515/cclm-2012-0228 23093336

[62] Kang D, Shi B, Erfe MC, Craft N, Li H. 2015. Vitamin B_12_ modulates the transcriptome of the skin microbiota in acne pathogenesis. Sci Transl Med 7:293ra103. doi:10.1126/scitranslmed.aab2009 PMC6049814 26109103

[63] Linhartová I, Bumba L, Mašín J, Basler M, Osička R, Kamanová J, Procházková K, Adkins I, Hejnová-Holubová J, Sadílková L, Morová J, Sebo P. 2010. RTX proteins: a highly diverse family secreted by a common mechanism. FEMS Microbiol Rev 34:1076–1112. doi:10.1111/j.1574-6976.2010.00231.x 20528947 PMC3034196

[64] Satchell KJF. 2015. Multifunctional-autoprocessing repeats-in-toxin (MARTX) toxins of vibrios. Microbiol Spectr 3. doi:10.1128/microbiolspec.VE-0002-2014 PMC4509488 26185092

[65] Lee C-T, Pajuelo D, Llorens A, Chen Y-H, Leiro JM, Padrós F, Hor L-I, Amaro C. 2013. MARTX of Vibrio vulnificus biotype 2 is a virulence and survival factor. Environ Microbiol 15:419–432. doi:10.1111/j.1462-2920.2012.02854.x 22943291

[66] Lo H-R, Lin J-H, Chen Y-H, Chen C-L, Shao C-P, Lai Y-C, Hor L-I. 2011. RTX toxin enhances the survival of Vibrio vulnificus during infection by protecting the organism from phagocytosis. J Infect Dis 203:1866–1874. doi:10.1093/infdis/jir070 21422475

[67] Tweedy JM, Park RWA, Hodgkiss W. 1968. Evidence for the presence of fimbriae (Pili) on Vibrio species. J Gen Microbiol 51:235–244. doi:10.1099/00221287-51-2-235 4870841

[68] Bessaiah H, Anamalé C, Sung J, Dozois CM. 2021. What flips the switch? Signals and stress regulating extraintestinal pathogenic Escherichia coli type 1 fimbriae (Pili). Microorganisms 10:5. doi:10.3390/microorganisms10010005 35056454 PMC8777976

[69] Crisan CV, Hammer BK. 2020. The Vibrio cholerae type VI secretion system: toxins, regulators and consequences. Environ Microbiol 22:4112–4122. doi:10.1111/1462-2920.14976 32133757

[70] Winzer K, Hardie KR, Williams P. 2003. LuxS and autoinducer-2: their contribution to quorum sensing and metabolism in bacteria, p 291–396. In Advances in applied microbiology. Academic Press. 10.1016/s0065-2164(03)53009-x 14696323

[71] Beeston AL, Surette MG. 2002. Pfs-dependent regulation of autoinducer 2 production in Salmonella enterica serovar Typhimurium. J Bacteriol 184:3450–3456. doi:10.1128/JB.184.13.3450-3456.2002 12057938 PMC135139

[72] Cadieux N, Bradbeer C, Reeger-Schneider E, Köster W, Mohanty AK, Wiener MC, Kadner RJ. 2002. Identification of the periplasmic cobalamin-binding protein BtuF of Escherichia coli. J Bacteriol 184:706–717. doi:10.1128/JB.184.3.706-717.2002 11790740 PMC139523

[73] DeVeaux LC, Kadner RJ. 1985. Transport of vitamin B_12_ in Escherichia coli: cloning of the btuCD region. J Bacteriol 162:888–896. doi:10.1128/jb.162.3.888-896.1985 2987192 PMC215858

[74] Di Girolamo PM, Bradbeer C. 1971. Transport of vitamin B _12_ in Escherichia coli*.* J Bacteriol 106:745–750. doi:10.1128/jb.106.3.745-750.1971 4934062 PMC248688

[75] Bansal T, Jesudhasan P, Pillai S, Wood TK, Jayaraman A. 2008. Temporal regulation of enterohemorrhagic Escherichia coli virulence mediated by autoinducer-2. Appl Microbiol Biotechnol 78:811–819. doi:10.1007/s00253-008-1359-8 18256823

[76] Higgins DA, Pomianek ME, Kraml CM, Taylor RK, Semmelhack MF, Bassler BL. 2007. The major Vibrio cholerae autoinducer and its role in virulence factor production. Nature New Biol 450:883–886. doi:10.1038/nature06284 18004304

[77] Cao M, Feng Y, Wang C, Zheng F, Li M, Liao H, Mao Y, Pan X, Wang J, Hu D, Hu F, Tang J. 2011. Functional definition of LuxS, an autoinducer-2 (AI-2) synthase and its role in full virulence of Streptococcus suis serotype 2. J Microbiol 49:1000–1011. doi:10.1007/s12275-011-1523-1 22203565

[78] Fong KP, Chung WO, Lamont RJ, Demuth DR. 2001. Intra- and interspecies regulation of gene expression by Actinobacillus actinomycetemcomitans LuxS. Infect Immun 69:7625–7634. doi:10.1128/IAI.69.12.7625-7634.2001 11705942 PMC98856

[79] Maharajan AD, Hjerde E, Hansen H, Willassen NP. 2022. Quorum sensing controls the CRISPR and type VI secretion systems in Aliivibrio wodanis 06/09/139. Front Vet Sci 9. doi:10.3389/fvets.2022.799414 PMC8861277 35211539

[80] Azimi S, Klementiev AD, Whiteley M, Diggle SP. 2020. Bacterial quorum sensing during infection. Annu Rev Microbiol 74:201–219. doi:10.1146/annurev-micro-032020-093845 32660382 PMC13064819

[81] Wang L, Chen Y, Huang H, Huang Z, Chen H, Shao Z. 2015. Isolation and identification of Vibrio campbellii as a bacterial pathogen for luminous vibriosis of Litopenaeus vannamei. Aquac Res 46:395–404. doi:10.1111/are.12191

[82] Haldar S, Chatterjee S, Sugimoto N, Das S, Chowdhury N, Hinenoya A, Asakura M, Yamasaki S. 2011. Identification of Vibrio campbellii isolated from diseased farm-shrimps from south India and establishment of its pathogenic potential in an artemia model. Microbiology (Reading) 157:179–188. doi:10.1099/mic.0.041475-0 20847009

