# Supplementary material for: Effects of vitamin B12 supply on cellular processes of the facultative vitamin B12 consumer Vibrio campbellii
Source: Appl Environ Microbiol. 2025 Jan 22;91(2):e01422-24. doi: 10.1128/aem.01422-24 (PMC11837498; doi:10.1128/aem.01422-24)
Supplement: Fig. S1 — Operon encoding pfs in the genome of V. campbellii, which is the first gene of the two-step AI-2 synthesis. [file aem.01422-24-s0003.pdf]

Supplementary Material Figure S1. The operon encoding pfs in the genome of V. campbellii, which is the first gene of the two-step AI-2 synthesis, as illustrated. The PFs, CbiB/CbiD, BtuF and YeiH genes/enzymes are shown in more detail, with information on enzyme name, EC number (if available) and protein sequence. Furthermore, similar operons (encoding the pfs gene) and their positioning in the genome are graphically depicted (IMG (https://img.jgi.doe.gov) with color scaffold set to COG) for all strains of the Vibrionales order.

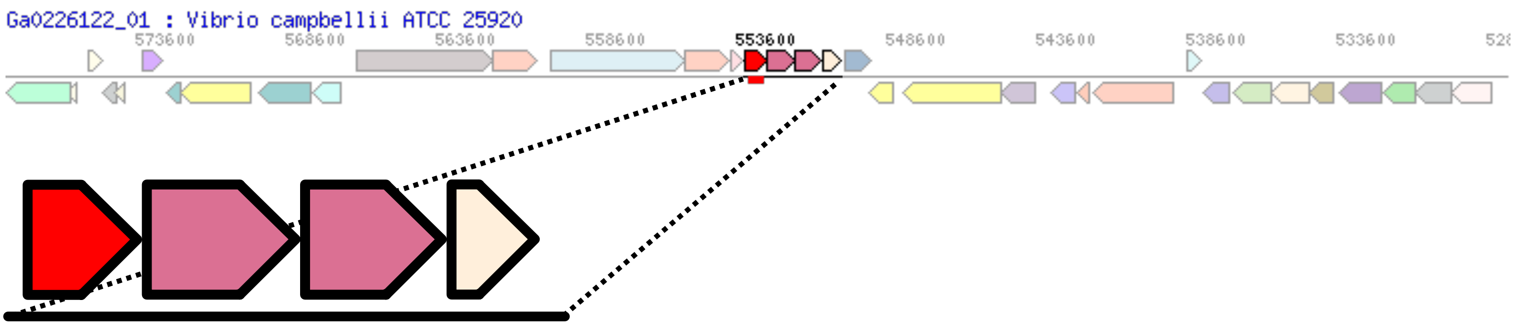

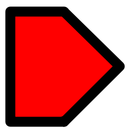

PFs - Adenosylhomocysteine nucleosidase [EC:3.2.2.9];
Autoinducer AI-2 biosynthesis I

(MKVGIIGAMEQEVTILKEAMTNCQTVSKAGCTFFSGQINDVDVVLLQSGIGKVAAAVGTTILLDEYQPDVVINTGSAGGFDSSLNLGDV
VISTEVRHHDADVTAFGYEMGQMAGQPAAFKADEKLMDLAEKALAQMENTHAVRGLICTGDAFVCTAERQEFIRKHFPSVIAVEMEASAI
AQTCHQFNTPFVVVRAISDVADKESPMSFEEFLPLAAKSSSEMVFKMLELTK)

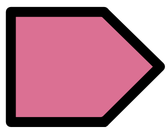

CbiB, CobD - Adenosylcobinamide-phosphate synthase [EC:6.3.1.10]
Adenosylcobalamin salvage from cobinamide II

(MEEIFQQLYANGALLVMWGALLFHLLIPIPHAAHPVTLWHKFAEQLANKVNNNHNYSQSILSGGLALFLMLVPCLILLLAMKPLVWQAPLFE
LALLLIALDWRNNESLAKQLVTAMSNENKAQCRALLKPYLNRDTESLSLLGIGKAGAETIIMGYGRNVVCVLFWYGFTGGIGALMYRLTAELA
RAWSPSRRQYSPFGKPAIQLTAALEIVPLRLFAVFISMGKNLSSVSTAMLQQAKSWPLPGPAWLLCSIGNKLQLSIGGPALYQGQRAERAKI
GGRIAPSAIHLAQIQTLIVWRIFVWIVIQSLILGLIYQGL)

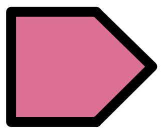

BtuF - Vitamin B_{12} transport system substrate-binding protein [EC:7.6.2.8]
Vitamin B_{12}-binding protein

(MMNKLCFALPLIFSAASFANEPAQRIISLAPHSTEIAYAAGLGDKLIAVSEMSDYPEQVKDLEKVSNYQGIKLERIIALQPDLVIAWPAGNPA
KELEKLEQFGTPIYYSTTGTLEDIATNLEQLSQYSEKPEVGQKAAAEFRAQLEALKEKYNTEDKVSYFYQLSEKPIITVAGKNWPSEVFTFCG
GENIFAKGSAPYPQVSIEQVITRQPEVLFASRHAMSNDGMWAEWKNDIPALRNNHVWSLHSDWINRPTPRTLNAITEVCEHFEAVRQKR)

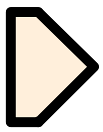

YeiH - Uncharacterized inner membrane protein

(MDSMLLYMIDLFGTAVFAVSGVLLAGRLKMDPFGVIVLGSVTAIGGGTIRDMALGATPVFWITDTTYLWVIFITCLLTMIVIRRPKRLPWWV
LPVCDAIGLAVFVGIGVEKALAYNASGMVAVIMGVITGCGGGIIRDVLAREVPMVLRSEVYATACIIGGIFHTTALSMGHDHSVALLAGVIST
LIIRLGAIRWHLSLPTFAINR)

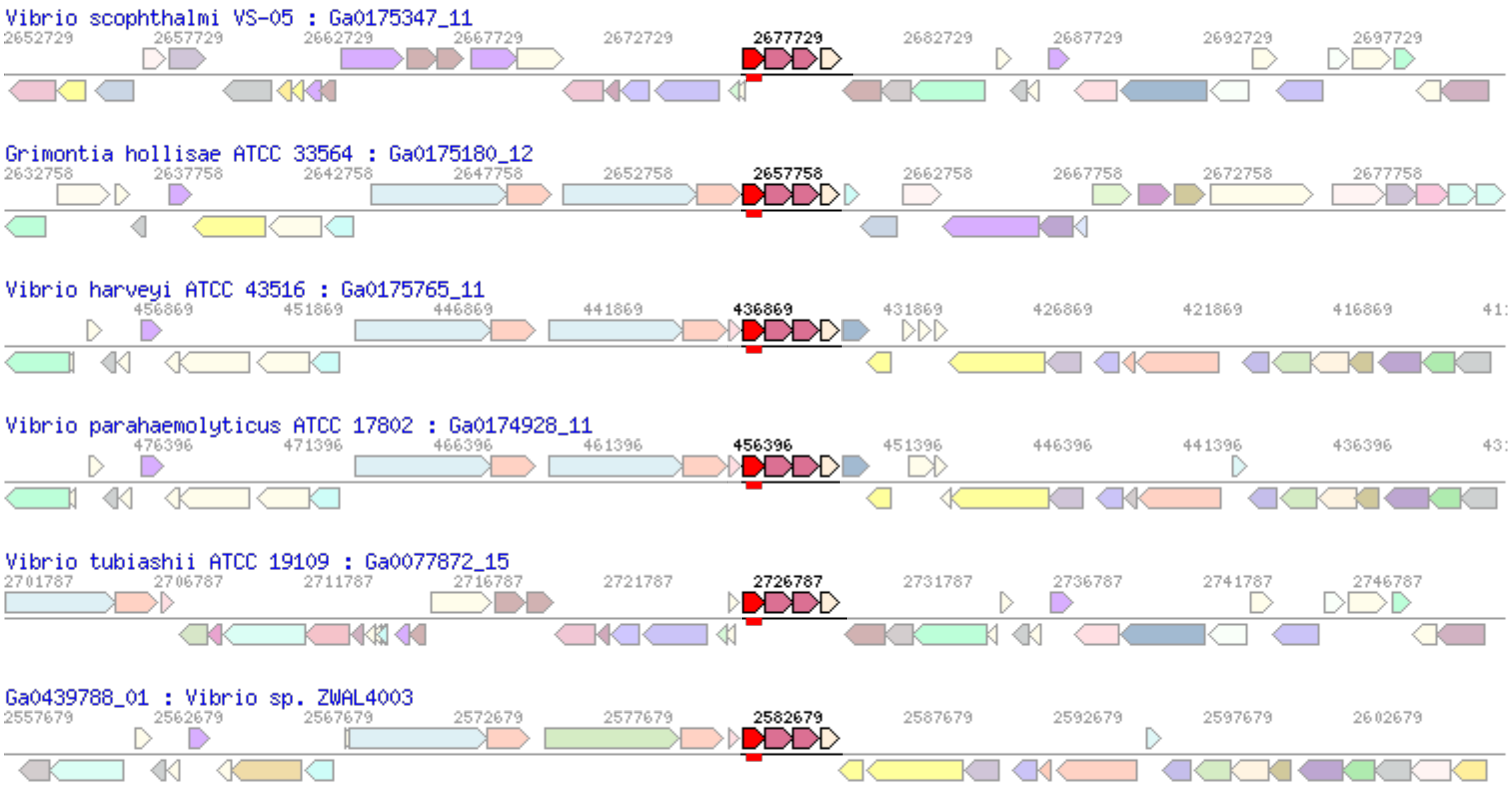

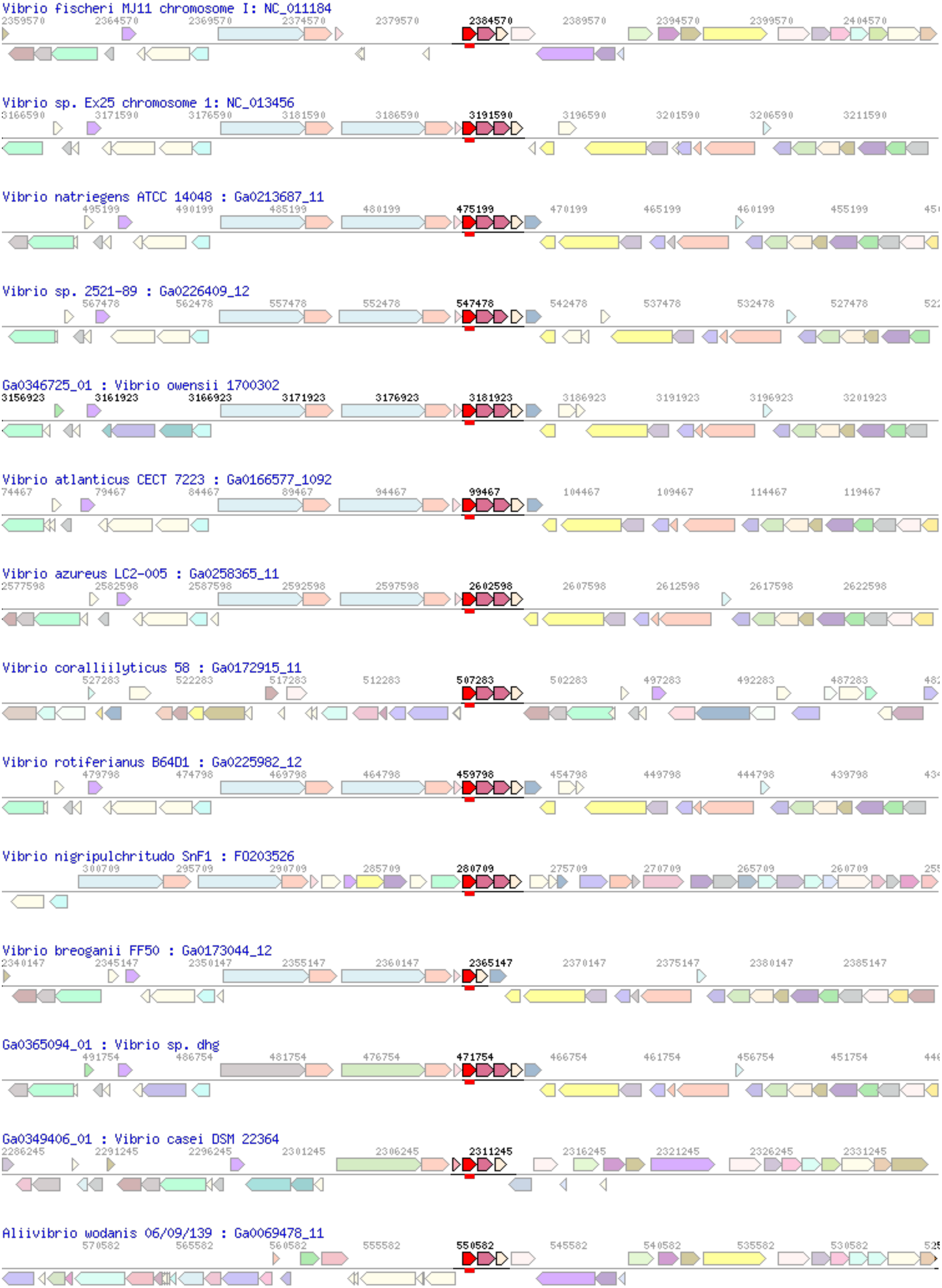

Vibrio fischeri MJ11 chromosome I: NC_011184
2359570 2364570 2369570 2374570 2379570 2384570 2389570 2394570 2399570 2404570
Vibrio sp. Ex25 chromosome 1: NC_013456
3166590 3171590 3176590 3181590 3186590 3191590 3196590 3201590 3206590 3211590
Vibrio natriegens ATCC 14048 : Ga0213687_11
495199 490199 485199 480199 475199 470199 465199 460199 455199
Vibrio sp. 2521-89 : Ga0226409_12
567478 562478 557478 552478 547478 542478 537478 532478 527478
Ga0346725_01 : Vibrio owensii 1700302
3156923 3161923 3166923 3171923 3176923 3181923 3186923 3191923 3196923 3201923
Vibrio atlanticus CECT 7223 : Ga0166577_1092
74467 79467 84467 89467 94467 99467 104467 109467 114467 119467
Vibrio azureus LC2-005 : Ga0258365_11
2577598 2582598 2587598 2592598 2597598 2602598 2607598 2612598 2617598 2622598
Vibrio coralliilyticus 58 : Ga0172915_11
527283 522283 517283 512283 507283 502283 497283 492283 487283
Vibrio rotiferianus B64D1 : Ga0225982_12
479798 474798 469798 464798 459798 454798 449798 444798 439798
Vibrio nigripulchritudo SnF1 : FO203526
300709 295709 290709 285709 280709 275709 270709 265709 260709
Vibrio breoganii FF50 : Ga0173044_12
2340147 2345147 2350147 2355147 2360147 2365147 2370147 2375147 2380147 2385147
Ga0365094_01 : Vibrio sp. dhg
491754 486754 481754 476754 471754 466754 461754 456754 451754
Ga0349406_01 : Vibrio casei DSM 22364
2286245 2291245 2296245 2301245 2306245 2311245 2316245 2321245 2326245 2331245
Aliivibrio wodanis 06/09/139 : Ga0069478_11
570582 565582 560582 555582 550582 545582 540582 535582 530582

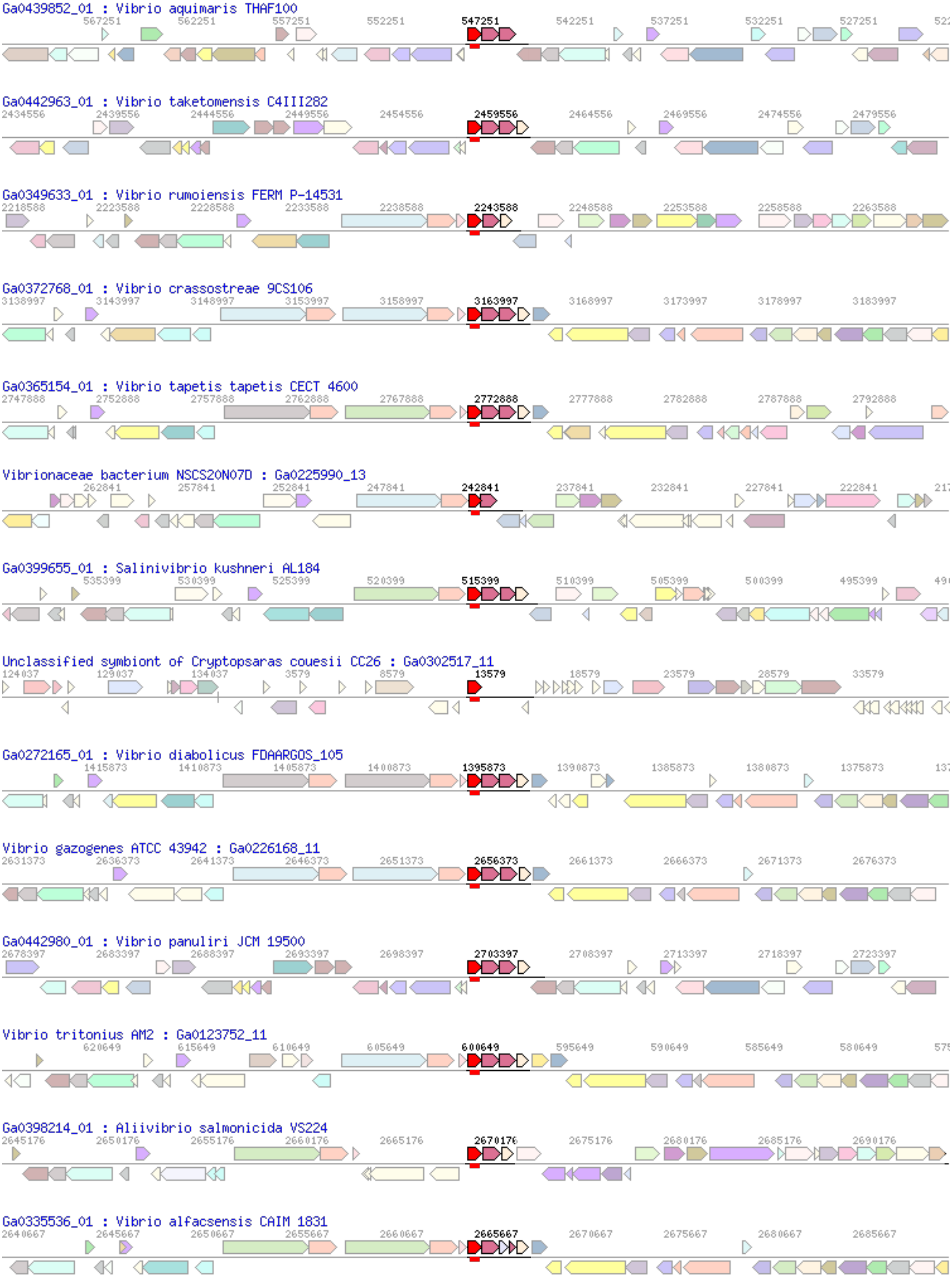
Ga0439852_01 : Vibrio aquimaris THAF100
567251 562251 557251 552251 547251 542251 537251 532251 527251
Ga0442963_01 : Vibrio taketomensis C4III282
2434556 2439556 2444556 2449556 2454556 2459556 2464556 2469556 2474556 2479556
Ga0349633_01 : Vibrio rumoiensis FERM P-14531
2218588 2223588 2228588 2233588 2238588 2243588 2248588 2253588 2258588 2263588
Ga0372768_01 : Vibrio crassostreae 9CS106
3138997 3143997 3148997 3153997 3158997 3163997 3168997 3173997 3178997 3183997
Ga0365154_01 : Vibrio tapetis tapetis CECT 4600
2747888 2752888 2757888 2762888 2767888 2772888 2777888 2782888 2787888 2792888
Vibrionaceae bacterium NSCS20N07D : Ga0225990_13
262841 257841 252841 247841 242841 237841 232841 227841 222841
Ga0399655_01 : Salinivibrio kushneri AL184
535399 530399 525399 520399 515399 510399 505399 500399 495399
Unclassified symbiont of Cryptopsaras couesii CC26 : Ga0302517_11
124037 129037 134037 3579 8579 13579 18579 23579 28579 33579
Ga0272165_01 : Vibrio diabolicus FDAARGOS_105
1415873 1410873 1405873 1400873 1395873 1390873 1385873 1380873 1375873
Vibrio gazogenes ATCC 43942 : Ga0226168_11
2631373 2636373 2641373 2646373 2651373 2656373 2661373 2666373 2671373 2676373
Ga0442980_01 : Vibrio panuliri JCM 19500
2678397 2683397 2688397 2693397 2698397 2703397 2708397 2713397 2718397 2723397
Vibrio tritonius AM2 : Ga0123752_11
620649 615649 610649 605649 600649 595649 590649 585649 580649
Ga0398214_01 : Aliivibrio salmonicida VS224
2645176 2650176 2655176 2660176 2665176 2670176 2675176 2680176 2685176 2690176
Ga0335536_01 : Vibrio alfacsensis CAIM 1831
2640667 2645667 2650667 2655667 2660667 2665667 2670667 2675667 2680667 2685667

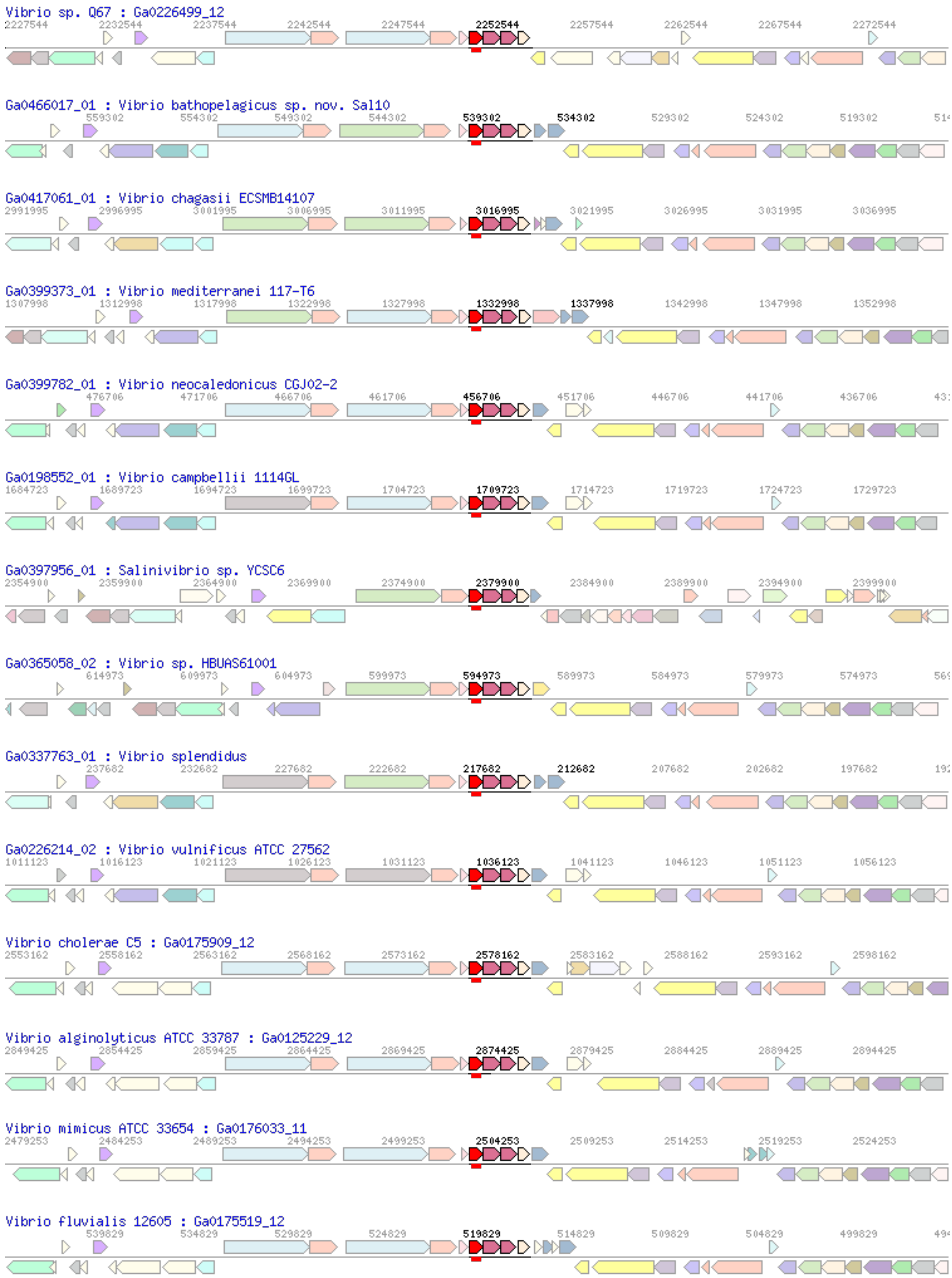
Vibrio sp. Q67 : Ga0226499_12
2227544 2232544 2237544 2242544 2247544 2252544 2257544 2262544 2267544 2272544
Ga0466017_01 : Vibrio bathopelagicus sp. nov. Sal10
559302 554302 549302 544302 539302 534302 529302 524302 519302
Ga0417061_01 : Vibrio chagasii ECSMB14107
2991995 2996995 3001995 3006995 3011995 3016995 3021995 3026995 3031995 3036995
Ga0399373_01 : Vibrio mediterranei 117-T6
1307998 1312998 1317998 1322998 1327998 1332998 1337998 1342998 1347998 1352998
Ga0399782_01 : Vibrio neocaledonicus CGJ02-2
476706 471706 466706 461706 456706 451706 446706 441706 436706
Ga0198552_01 : Vibrio campbellii 1114GL
1684723 1689723 1694723 1699723 1704723 1709723 1714723 1719723 1724723 1729723
Ga0397956_01 : Salinivibrio sp. YCSC6
2354900 2359900 2364900 2369900 2374900 2379900 2384900 2389900 2394900 2399900
Ga0365058_02 : Vibrio sp. HBUAS61001
614973 609973 604973 599973 594973 589973 584973 579973 574973
Ga0337763_01 : Vibrio splendidus
237682 232682 227682 222682 217682 212682 207682 202682 197682
Ga0226214_02 : Vibrio vulnificus ATCC 27562
1011123 1016123 1021123 1026123 1031123 1036123 1041123 1046123 1051123 1056123
Vibrio cholerae C5 : Ga0175909_12
2553162 2558162 2563162 2568162 2573162 2578162 2583162 2588162 2593162 2598162
Vibrio alginolyticus ATCC 33787 : Ga0125229_12
2849425 2854425 2859425 2864425 2869425 2874425 2879425 2884425 2889425 2894425
Vibrio mimicus ATCC 33654 : Ga0176033_11
2479253 2484253 2489253 2494253 2499253 2504253 2509253 2514253 2519253 2524253
Vibrio fluvialis 12605 : Ga0175519_12
539829 534829 529829 524829 519829 514829 509829 504829 499829
