# Supplementary material for: Effects of vitamin B12 supply on cellular processes of the facultative vitamin B12 consumer Vibrio campbellii
Source: Appl Environ Microbiol. 2025 Jan 22;91(2):e01422-24. doi: 10.1128/aem.01422-24 (PMC11837498; doi:10.1128/aem.01422-24)
Supplement: Supplemental legends — Legends for supplemental material. [file aem.01422-24-s0004.docx]

**Supplementary Material Legends (Figure, Data and Table)**

**Supplementary Material Figure S1.** The operon encoding *pfs* in the genome of *V. campbellii*, which is the first gene of the two-step AI-2 synthesis, is illustrated. The PFs, CbiB/CbiD, BtuF and YeiH genes/enzymes are shown in more detail, with information on enzyme name, EC number (if available) and protein sequence. Furthermore, similar operons (encoding the *pfs* gene) and their positioning in the genome are graphically depicted (IMG (<https://img.jgi.doe.gov>) with color scaffold set to COG) for all strains of the *Vibrionales* order.

**Supplementary Material Table S1.** Extra- and intracellular B_12_ (Cyanocobalamin, Adenosylcobalamin, Methylcobalamin, Hydroxycobalamin) and activated lower ligand (α-ribazole) recovery by LC-MS in *V. campbellii* cultures.

**Supplementary Material Table S2.** Growth time, yield and rate of *V. campbellii* determined by optical density when growing in mono-culture with (1 nM) and without B_12_ supplementation or the addition of methionine (10 µM) or cobinamide (1 nM).

**Supplementary Material Data S1.** Listed are 3716 bacterial genomes that are publicly available on https://img.jgi.doe.gov/, that possess at least 54 of the 55 surveyed housekeeping genes. Gene presence of the B_12_ pathway and the activated methyl cycle for each surveyed bacterial stain are listed here and graphically summarized and depicted in Figure 1. Moreover, highlighted are genomes of the gammaproteobacteria class and the order of *Vibrionales*.

**Supplementary Material Data S2.** Transcriptional gene regulation of *V. campbellii* culture when supplemented with and without the addition of B_12_ (1 nM). Genes transcribed at a log-fold-change below -1.5 and above 1.5 are outlined in separate sheets, highlighting respective cellular functions. The statistical relevance is given as the adj. *p*-value.
