# Supplementary material for: Effects of vitamin B12 supply on cellular processes of the facultative vitamin B12 consumer Vibrio campbellii
Source: Appl Environ Microbiol. 2025 Jan 22;91(2):e01422-24. doi: 10.1128/aem.01422-24 (PMC11837498; doi:10.1128/aem.01422-24)
Supplement: Table S1 — Extra- and intracellular B12 (cyanocobalamin, adenosylcobalamin, methylcobalamin, hydroxycobalamin) and activated lower ligand (α-ribazole) recovery by LC-MS in V. campbellii cultures. [file aem.01422-24-s0005.docx]

**Supplementary Material Table S1.** Extra- and intracellular B_12_ (Cyanocobalamin, Adenosylcobalamin, Methylcobalamin, Hydroxycobalamin) and activated lower ligand (α-ribazole) recovery by LC-MS in *V. campbellii* cultures.

| Vitamin | SPE method  (Extracellular) | Recovery (%)  Particulate extraction method  (Intracellular) | Limit of detection SPE (pM) |
| --- | --- | --- | --- |
| Cyanocobalamin (CB_12_) | 91 | 99 | 0.08 |
| Adenosylcobalamin (AB_12_) | 65 | 94 | 0.10 |
| Methylcobalamin (MB_12_) | 59 | 97 | 0.05 |
| Hydroxycobalamin (HB_12_) | 48 | 74 | 6.15 |
| Alpha-ribazole (α-Rib) | 71 | 97 | 0.01 |
