# Supplementary material for: Effects of vitamin B12 supply on cellular processes of the facultative vitamin B12 consumer Vibrio campbellii
Source: Appl Environ Microbiol. 2025 Jan 22;91(2):e01422-24. doi: 10.1128/aem.01422-24 (PMC11837498; doi:10.1128/aem.01422-24)
Supplement: Table S2 — Growth time, yield, and rate of V. campbellii determined by optical density when growing in mono-culture with (1 nM) and without B12 supplementation or the addition of methionine (10 µM) or cobinamide (1 nM). [file aem.01422-24-s0006.docx]

**Supplementary Material Table S2.** Growth time, yield and rate of *V. campbellii* determined by optical density when growing in mono-culture with (1 nM) and without B_12_ supplementation or the addition of methionine (10 µM) or cobinamide (1 nM).

| **Treatment** | **Hour** | **Growth yield (OD600)** | **SD**  **(Growth yield)** | **Growth rate (h^-1^)** |
| --- | --- | --- | --- | --- |
| Vitamin B_12_ (1 nM) | 77 | 0.334 | 0.028 | 0.043 |
| Methionine | 82 | 0.254 | 0.024 | 0.035 |
| Cobinamide | 92 | 0.288 | 0.013 | 0.041 |
| No addition | 96 | 0.257 | 0.013 | 0.041 |
